# Disruption of Mitochondrial Dynamics and Integrity Drives Divergent Metabolic Flexibility and Resilience in Podocytes

**DOI:** 10.1096/fj.202502934R

**Published:** 2025-12-13

**Authors:** Cem Özel, Katrin M. Reitmeier, Emilia Kieckhöfer, Khawla Abualia, Duc Nguyen‐Minh, Mahsa Matin, Henning Hagmann, Richard J. M. Coward, Sebastian Brähler, Philipp Antczak, Bernhard Schermer, Thomas Benzing, Patrick Giavalisco, Paul T. Brinkkoetter

**Affiliations:** ^1^ Department II of Internal Medicine and Center for Molecular Medicine Cologne, Faculty of Medicine and University Hospital Cologne University of Cologne Cologne Germany; ^2^ Bristol Renal, Bristol Medical School, Faculty of Health Sciences University of Bristol Bristol UK; ^3^ Cluster of Excellence Cellular Stress Response in Aging‐Associated Diseases (CECAD), Faculty of Medicine and University Hospital Cologne, University of Cologne Cologne Germany; ^4^ Max Planck Institute for Biology of Aging Cologne Germany

**Keywords:** anaplerosis, glycolysis, insulin signaling, metabolism, mitochondria, OMA1, PHB2, podocytes

## Abstract

Mitochondrial dysfunction is central to the pathogenesis of podocytopathies, yet the determinants of metabolic resilience versus failure remain elusive. We investigated how distinct disruptions of mitochondrial architecture, specifically hyperfusion via OMA1 deletion versus compromised inner mitochondrial membrane (IMM) integrity via PHB2 knockdown, influence the metabolic fate and insulin responsiveness of podocytes. To this end, we analyzed conditionally immortalized mouse podocytes with genetic OMA1 deletion or inducible PHB2 knockdown and employed an integrated approach combining bioenergetic studies, quantitative proteomics, phosphoproteomics, metabolomics, and stable isotope tracing studies with ^13^C_6_‐glucose and ^13^C_5_‐glutamine. We characterized metabolic remodeling at baseline and after insulin treatment and uncovered profoundly divergent metabolic states. OMA1 deficiency conferred robust metabolic resilience, characterized by a compensatory glycolytic shift and remodeling of TCA cycle flux through glutamine‐driven anaplerosis while maintaining oxidative phosphorylation. OMA1‐deficient podocytes sustained bioenergetic homeostasis upon insulin challenge by flexibly rerouting carbon flux, including the GABA shunt. In contrast, PHB2 deficiency led to metabolic failure, impaired respiration, and anaplerotic insufficiency. While maintaining basal ATP levels at baseline, PHB2‐deficient podocytes exhibited energetic collapse upon insulin treatment, revealing profound metabolic inflexibility. Taken together, the structural integrity of the inner mitochondrial membrane, rather than mitochondrial morphology per se, is a driving determinant of metabolic competence and resilience in podocytes.

## Graphical abstract

We investigated two models of mitochondrial dysfunction in podocytes, using two cell culture models: podocytes isolated from mice with Oma1 deletion (Oma1^del^) and mouse podocytes modified with an inducible knockdown of PHB2 (Phb2^kd^). OMA1 deficiency induced a glycolytic shift, enhanced glutamine anaplerosis, and sustained energy homeostasis after metabolomic challenging with insulin. In contrast, PHB2 deficiency disrupted mitochondrial inner membrane integrity, impaired respiration, and led to anaplerotic insufficiency, with energetic collapse upon metabolomic challenging with insulin.

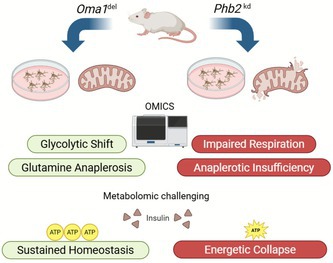

## 1 Introduction

Podocytes are highly specialized kidney cells that maintain the selective permeability of the glomerular filtration barrier through interdigitating foot processes connected by slit diaphragms [1]. Disruption of this barrier leads to nephrotic syndrome, characterized by proteinuria, hypoalbuminemia, hyperlipidemia, and edema, due to the unregulated passage of macromolecules into the urine [2]. Although genetic mutations, metabolic disorders, and chemical stressors all contribute to glomerular disease, most cases that progress to end‐stage renal disease share a common histological pattern, that is, focal segmental glomerulosclerosis (FSGS) [3, 4]. While injury to any glomerular component, podocytes, endothelial cells, or the glomerular basement membrane, can result in chronic kidney disease, the degree of podocyte injury and, ultimately, loss of podocytes is the predominant driver in the majority of glomerulopathies [5].

Insulin signaling is critical for preserving podocyte function and glomerular barrier integrity [6]. Upon insulin binding, its receptor activates PI3K/AKT signaling, which regulates glucose uptake, cytoskeletal organization, and cell survival [7]. AKT activation, in turn, stimulates mTORC1, a nutrient‐sensitive kinase complex essential for podocyte metabolism and growth [8, 9]. The necessity for precise mTORC1 regulation has been demonstrated in various glomerular diseases, including FSGS, where both hyperactivation and suppression of mTORC1 have been linked to podocyte damage [10, 11, 12, 13, 14, 15, 16]. Pharmacological inhibition of mTORC1 with rapamycin has shown promise in restoring signaling balance and reducing podocyte apoptosis, particularly under high‐glucose conditions [17]. Moreover, insulin resistance in podocytes disrupts nephrin phosphorylation, a key event for maintaining slit diaphragm integrity and GLUT4 trafficking, leading to impaired glucose uptake and cytoskeletal remodeling [18, 19].

Mitochondrial dysfunction is increasingly recognized as a contributing factor in podocytopathies such as diabetic kidney disease and FSGS [20]. Excessive production of reactive oxygen species and impaired mitophagy exacerbate cellular injury in these contexts. Mitochondria continuously adapt to cellular demands via dynamic processes of fusion and fission, regulated in part by proteolytic processing of the GTPase OPA1 [21, 22]. Prohibitins (PHB1 and PHB2) form ring‐like structures in the inner mitochondrial membrane, stabilizing membrane architecture and forming lipid microdomains [23]. Within these domains, the metallopeptidase OMA1 is activated under stress conditions, cleaving long OPA1 isoforms (L‐OPA1) into short forms (S‐OPA1), thereby inhibiting fusion and promoting mitochondrial fragmentation [24, 25, 26, 27].

In podocytes, mitochondria are primarily localized in the central cell body and primary processes [28]. Energy production is highly compartmentalized, with glycolysis supporting the dynamic remodeling of peripheral foot processes and oxidative phosphorylation meeting the overall high energy demands, particularly under metabolic stress [29]. In parallel, glutaminolysis can sustain energy production during glucose deprivation by fueling anaplerotic flux into the TCA cycle, converting glutamine to α‐ketoglutarate [30]. To explore how mitochondrial dynamics influence podocyte metabolism and signaling, we employed two complementary models disrupting mitochondrial dynamics and integrity: podocytes with genetic deletion of OMA1 and knockdown of PHB2. Importantly, neither model directly disrupts the electron transport chain, but both selectively alter mitochondrial morphology through effects on fusion and fission. OMA1‐depleted cells preserve unprocessed OPA1 and favor mitochondrial fusion, while PHB2‐deficient cells destabilize the inner mitochondrial membrane, leading to excessive fission. We assessed mitochondrial and cellular energetics using Seahorse assays, characterized global metabolic changes through metabolomics and proteomics, and mapped metabolic pathway utilization using ^13^C‐labeled glucose and glutamine tracing under both basal and insulin‐stimulated conditions. Given the central role of insulin sensitivity and mTORC1 signaling in podocyte health, we utilized insulin stimulation to probe metabolic flexibility and rapamycin to investigate the impact of mTORC1 modulation under conditions of mitochondrial dysfunction. Our goal was to define how these distinct disruptions of mitochondrial homeostasis reprogram podocyte metabolism and to identify metabolic vulnerabilities or adaptive mechanisms that could serve as therapeutic targets in glomerular disease.

## 2 Materials and Methods

### 2.1 Cell Culture

Conditionally immortalized heat‐sensitive mouse podocytes (HSMP) expressing tetracycline‐inducible PHB2 shRNA constructs were established via lentiviral transduction of wild‐type HSMP using pLenti4/TO plasmids, as previously described, while cells transduced with a tetracycline‐inducible scrambled shRNA served as controls [31]. PHB2 knockdown was induced by adding 0.1 μg/mL doxycycline for 48 h prior to assays. Heat‐sensitive *Oma1* ^del^ podocytes were derived from kidney isolates of *Oma1* ^del^ mice (*Oma1* ^fl/fl^; CMV:Cre^tg/wt^), as previously described, while podocytes from wild‐type mice served as controls [32]. Care and experimental protocols were in accordance with national guidelines and approved by the University of Cologne Animal Care Committee and local government authorities (LANUV NRW). Podocytes were maintained in RPMI 1640 medium supplemented with 10% fetal bovine serum, 5% SPS (100 mM), and 5% Hepes buffer (1 M, pH 7.4) (Gibco/Thermo Fisher Scientific, Waltham, MA, USA). Cells were grown at 33°C under permissive conditions and shifted to 37°C for 10 days to induce differentiation before experiments. For inhibitor studies, rapamycin (Sigma, #R8781) was added at 10 ng/mL for 2 h. For insulin stimulation, podocytes were serum‐starved in FBS‐free medium overnight, then treated with 10 μg/mL insulin (ITS; Corning) for 3 h. For stable isotope tracing, cells were incubated overnight in RPMI lacking either glucose (Gibco, #11879020) or glutamine (Gibco, #21870076). The following day, U^13^C_6_ glucose (Eurisotop, #389374‐1G) or U^13^C_5_ glutamine (Eurisotop, #CLM‐1822‐H‐0.1) was added 2 h prior to harvest. All experimental conditions were performed with at least three independent biological replicates.

### 2.2 Immunoblotting

Cells were harvested by scraping and lysed on ice in lysis buffer containing 1% Triton X‐100, 50 mM Tris‐HCl (pH 7.4), 150 mM NaCl, 50 mM NaF, and 15 mM sodium pyrophosphate (Na_4_P_2_O_7_), supplemented with sodium orthovanadate (Na₃VO₄) and protease/phosphatase inhibitors (PMSF and PIM; Sigma‐Aldrich, St. Louis, MO, USA). Protein concentrations were determined using the BCA Protein Assay Kit (Thermo Fisher Scientific, Waltham, MA, USA). Equal amounts of protein (30 μg) were mixed with Laemmli buffer, boiled at 95°C for 5 min, resolved by SDS–PAGE, and transferred to PVDF membranes (Millipore). Membranes were blocked and incubated overnight at 4°C with the indicated primary antibodies, followed by HRP‐conjugated secondary antibodies for 1 h at room temperature. Immunoreactive bands were visualized using SuperSignal West Femto ECL substrate (Thermo Fisher Scientific) and imaged with a Fusion Solo system. The following primary antibodies were used: rabbit anti‐PHB2 (1:1000, BioLegend, #611802), mouse anti‐OMA1 (1:1000, Santa Cruz Biotechnology, #sc515788), mouse anti‐β‐tubulin (1:1000, Sigma‐Aldrich, #T0198), rabbit anti‐phospho‐S6 ribosomal protein (Ser235/236; 1:1000, Cell Signaling Technology, #4858), and rabbit anti‐S6 ribosomal protein (1:1000, CST, #2217).

### 2.3 Electron Microscopy

Cultured podocytes were fixed (4% paraformaldehyde, 2% glutaraldehyde in 0.1 M sodium cacodylate, pH 7.4) for 30 min at room temperature and 30 min at 4°C. Samples were postfixed in 1% osmium tetroxide in 0.1 M cacodylate buffer, dehydrated through a graded ethanol series, and embedded in epoxy resin (Sigma‐Aldrich) according to the manufacturer's instructions. Ultrathin sections (30 nm) were stained with 1.5% aqueous uranyl acetate and lead citrate and subsequently examined using a Zeiss EM902 transmission electron microscope. Acquired images were processed in Adobe Photoshop CS4, and quantitative ultrastructural analysis was performed using Fiji/ImageJ in a blinded fashion [33].

### 2.4 Cellular Energetics

Cellular oxygen consumption (OCR) and extracellular acidification (ECAR) were measured using the Seahorse XFp Analyzer (Agilent Technologies, Santa Clara, CA, USA). Oxidative phosphorylation (OXPHOS) and glycolytic flux were assessed using the Mito Stress Test and Glycolysis Stress Test Kits, respectively, following the manufacturer's protocols. Briefly, 40 000 cells per well were seeded in XFp cell culture 96‐well plates and differentiated for 10 days. On the day of the assay, culture medium was replaced with Seahorse DMEM (Agilent, #103575) supplemented with 10 mM glucose, 2 mM glutamate, and 1 mM sodium pyruvate for OXPHOS measurements, or with 2 mM glutamate for glycolysis measurements, and incubated at 37°C for 45 min. For the Mito Stress Test, each well received sequential injections of 1 μM oligomycin, 2 μM FCCP, and 1 μM rotenone/antimycin A. For the Glycolysis Stress Test, 10 mM glucose, 1 μM oligomycin, and 50 mM 2‐deoxyglucose (2‐DG) were applied. Data were normalized to protein concentration, as determined by a BCA Protein Assay Kit (Thermo Fisher Scientific, Waltham, MA, USA), and analyzed using Agilent Wave software.

### 2.5 Metabolite Extraction

Cell monolayers were rapidly washed with 75 mM ammonium carbonate (pH 7.4) and flash‐frozen on liquid nitrogen. For metabolite extraction, cells were processed in two sequential steps using 2 × 800 μL of pre‐cooled (−20°C) extraction buffer composed of UPLC‐grade methanol, acetonitrile, and water in a 2:2:1 (v:v:v) ratio (Biosolve, Valkenswaard, Netherlands). For unlabeled samples, isotope‐labeled internal standards—comprising a mixture of ^13^C^15^N‐labeled amino acids, Cambridge Isotopes, MSK‐A2‐1.2 and ^13^C_10_ ATP, ^15^N_5_ ADP, ^13^C_10_^15^N_5_ AMP—and citric acid D4 (Sigma Aldrich)—were added. In the first extraction, plates were incubated at −20°C for 10 min, after which cells were scraped and the entire mixture, including precipitated material, was transferred to 2 mL Eppendorf tubes kept on ice. The residual cellular material was extracted a second time with an additional 800 μL of pre‐cooled extraction buffer; the resulting extract was pooled with the initial 800 μL to yield approximately 1600 μL total. The combined extracts were incubated for 30 min at 4°C, then centrifuged at 21000 × *g* for 10 min at 4°C to pellet proteins and insoluble debris. The cleared supernatant was divided into two 800 μL fractions, transferred into fresh 1.5 mL Eppendorf tubes, and immediately dried using a vacuum concentrator (Scan Speed 40, Labogene, Denmark). The resulting metabolite pellets were either stored at −80°C or analyzed directly by liquid chromatography–mass spectrometry.

### 2.6 nLC‐MS/MS

Cell lysates were prepared for proteomic analysis using standard methods [34, 35]. Briefly, cells were lysed in a buffer composed of 8 M urea, 50 mM ammonium bicarbonate, and protease/phosphatase inhibitors (Thermo Fisher Scientific, Waltham, MA, USA). Chromatin was sheared using an Athena Ultrasonic Probe Sonicator (Athena Technologies, Mumbai, IND), and lysates were clarified by centrifugation at 16 000 × *g* for 45 min at 4°C. Protein concentrations were determined using a BCA Protein Assay Kit (Thermo Fisher Scientific). For each sample, 800 μg of total protein was reduced with 5 mM dithiothreitol (DTT) for 60 min and subsequently alkylated with 20 mM iodoacetamide for an additional 60 min. The samples were then diluted 1:4 with 50 mM ammonium bicarbonate, and trypsin was added at a 1:100 ratio for overnight digestion. Digestion was halted the following day by adding 100% formic acid at a ratio of 1:200, and the samples were clarified by centrifugation at 16 000 × *g* for 10 min at room temperature. Peptide purification was carried out using Oasis HLB 1 cc/30 mg columns (Waters Corp., Milford, MA, USA) according to the manufacturer's instructions. Before drying the samples in a speedvac, an aliquot containing 30 μg of total protein was set aside and stored at −20°C for subsequent proteome analysis. Phosphopeptides were enriched from the peptide mixtures using an Fe‐NTA Phosphopeptide Enrichment Kit with immobilized metal affinity chromatography columns (Thermo Fisher Scientific), following the manufacturer's instructions. The enriched phosphopeptides were dried in a speedvac and resuspended in 0.1% formic acid. Peptide analysis was performed using an LTQ Orbitrap Discovery mass spectrometer (Thermo Fisher Scientific) coupled to a Proxeon EASY‐nLC II nano‐LC system (Thermo Fisher Scientific), operating at a flow rate of 250 nL/min over a 150‐min gradient [34]. MS1 survey scans were acquired over an m/z range of 300–2000 at a resolution of 70 000, and the top 10 most intense ions were selected within a 2 Da isolation window for higher‐energy collisional dissociation (HCD) fragmentation, as previously described [36]. Four biological replicates were analyzed for each experimental group.

### 2.7 Anion‐Exchange Chromatography High Resolution Mass Spectrometry (AEX‐HRMS)

For anionic metabolite analysis, dried extracts were reconstituted in 250 μL of UPLC/MS‐grade water (Biosolve, Valkenswaard, Netherlands), and 150 μL of each reconstituted sample was transferred into polypropylene autosampler vials (Chromatography Accessories Trott, Germany) prior to AEX‐MS analysis. Chromatographic separation was performed on a Dionex ion chromatography system (Integrion, Thermo Fisher Scientific). In brief, 5 μL of the polar extract was injected in push partial mode—with an overfill factor of 3—onto a Dionex IonPac AS11‐HC column (2 mm × 250 mm, 4 μm; Thermo Fisher Scientific) equipped with a Dionex IonPac AG11‐HC guard column (2 mm × 50 mm, 4 μm; Thermo Fisher Scientific). The column temperature was maintained at 30°C while the autosampler was held at 6°C. A potassium hydroxide gradient was generated on‐line using a KOH cartridge (Eluent Generator, Thermo Scientific) supplied with deionized water (Milli‐Q, IQ 700, Millipore). Metabolite separation was achieved at a flow rate of 380 μL/min under the following gradient conditions: 0–3 min at 10 mM KOH; 3–12 min, linear ramp from 10 mM to 50 mM KOH; 12–19 min, ramp from 50 mM to 100 mM KOH; 19–22 min at 100 mM KOH; 22–23 min, a linear decrease from 100 mM to 10 mM KOH; and a brief re‐equilibration at 10 mM KOH for 3 min. Eluting compounds were detected in negative ion mode via full scan on a Q‐Exactive HF high‐resolution mass spectrometer (Thermo Fisher Scientific) over an m/z range of 77–770. The heated electrospray ionization (ESI) source was operated with a spray voltage of 3.2 kV, a capillary temperature of 300°C, a sheath gas flow of 50 AU, an auxiliary gas flow of 20 AU at 330°C, and a sweep gas flow of 2 AU, with the S‐lens set at 60.

### 2.8 Ultra‐Performance Liquid Chromatography‐High‐Resolution Mass Spectrometry (UPLC‐HRMS)

For amine‐containing metabolite analysis, 50 μL of the previously obtained polar phase (from AEX‐HRMS) was mixed with 25 μL of 100 mM sodium carbonate (Sigma), followed by the addition of 25 μL of 2% (v/v) benzoyl chloride (Sigma) in acetonitrile (UPLC/MS‐grade, Biosolve, Valkenswaard, Netherlands). The resulting derivatized samples were mixed thoroughly by pipetting and maintained at room temperature (20°C) until analysis. For UPLC‐HRMS, 2 μL of each derivatized sample was injected onto a 100 × 2.1 mm HSS T3 UPLC column (Waters) at a flow rate of 400 μL/min using a binary solvent system. Buffer A consisted of 10 mM ammonium formate (Sigma) with 0.15% (v/v) formic acid (Sigma) in UPLC/MS‐grade water (Biosolve), and Buffer B was acetonitrile containing 0.1% formic acid (UPLC/MS‐grade, Biosolve). The column temperature was held at 40°C, and the following gradient was applied: 0%–15% B 0–4.1 min; 15%–17% B 4.1–4.5 min; 17%–55% B 4.5–11 min; 55%–70% B 11–11.5 min; 70%–100% B 11.5–13 min; 100% B 13–14 min; 100%–0% B 14–14.1 min; 0% B 14.1–19 min. Detection was performed using a Q‐Exactive Plus mass spectrometer (Thermo Fisher Scientific) operated in positive ion mode over an m/z range of 100–1000. The heated electrospray ionization (ESI) source settings were as follows: spray voltage, 3.5 kV; capillary temperature, 300°C; sheath gas flow, 60 AU; auxiliary gas flow, 20 AU at 330°C; sweep gas flow, 2 AU; and the RF‐lens was set to 60.

### 2.9 Bioinformatic Analysis (nLC‐MS/MS)

Raw mass spectrometry files were processed using MaxQuant Suite (v1.5.0.1) with label‐free quantification and match‐between‐runs enabled, searching against the mouse UniProt reference database. A mass accuracy threshold of 20 ppm was applied with deisotoping enabled and a mass tolerance set to 0.5 Da. Carbamidomethylation of cysteines was specified as a fixed modification, while variable modifications included phosphorylation on serine, threonine, and tyrosine residues, and methionine oxidation, with a maximum of four modifications allowed per peptide. Protein, peptide, and site false discovery rates were set to 0.1 by default. LFQ and phosphorylation site intensities were log‐transformed, potential contaminants were removed, and data were normalized by subtracting the mean. Gene Ontology terms were annotated using Perseus 1.5, and categorical one‐dimensional enrichment analysis was performed via Fisher's exact test.

### 2.10 Bioinformatic Analysis (AEX‐HRMS and UPLC‐HRMS)

Semi‐targeted raw data from both AEX‐HRMS and UPLC‐HRMS analyses were processed using TraceFinder 5.1 (Thermo Fisher Scientific). Compound identities were confirmed by comparison with authentic reference standards measured at the start and end of each sample set. For relative quantification of unlabeled analytes, the area of the monoisotopic deprotonated ([M–H]^−1^) or doubly deprotonated ([M–2H]^−2^) mass peak (M_0_) was extracted and integrated with a mass accuracy of < 3 ppm and a retention time tolerance of < 0.05 min relative to the reference compounds. The relative isotopic distribution was determined by calculating the fraction of each isotopologue's peak area relative to the sum of all isotopologues for that compound. Absolute ^13^C‐enrichment—defined as the proportion of ^13^C‐labeled molecules in each compound—was calculated by multiplying the peak area of each detected isotopologue by the ratio of ^13^C to ^12^C atoms in that isotopologue, and then summing these values to obtain the total ^13^C area. Dividing this total by the combined peak area of all isotopologues yielded the relative ^13^C enrichment factor. An analogous procedure was employed for the UPLC‐HRMS analysis of benzoylated (Bz) samples, where peak areas of [M + nBz + H]^+^ ions—nBz representing the number of benzoyl groups (C_7_H_4_O) attached—were extracted using the same mass (< 3 ppm) and retention time (< 0.05 min) criteria. Cellular pool sizes, isotopic distributions, and ^13^C enrichments were then computed as described for the AEX‐MS data, with corrections for natural ^13^C abundance applied and ^13^C signals normalized to the corresponding ^12^C pool size [37].

### 2.11 Visualization and Statistical Analysis

Visualizations were generated using Adobe Photoshop, Adobe Illustrator, and GraphPad Prism 9. Data are presented as means ± SEM unless stated otherwise. Statistical analyses were performed with GraphPad Prism 9. Data are presented as means ± SEM. Comparisons between two groups were performed using the unpaired Student's *t*‐test. Due to the inherent variability frequently observed in metabolomic datasets, Welch's t‐test was utilized when variances were found to be significantly different between groups. Significance levels were defined as *p < 0.05, **p < 0.01, ***p < 0.001, and ****p < 0.0001. For proteomic analyses, a false discovery rate (FDR) of less than 0.05 was considered significant.

## 3 Results

### 3.1 Disruption of Mitochondrial Dynamics and Integrity Drives Divergent Glycolytic Adaptations in Podocytes

**FIGURE 1:**
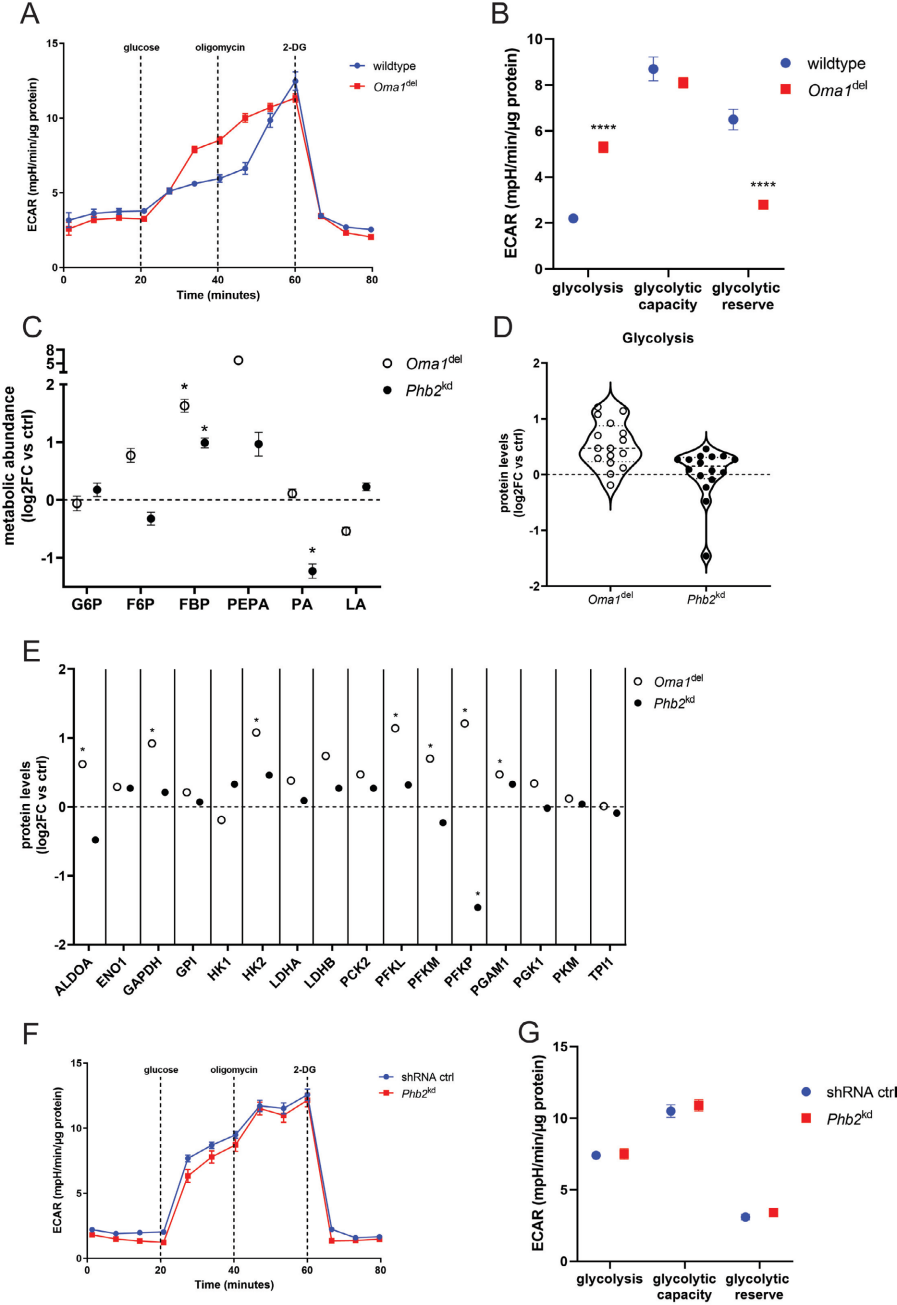
Disruption of mitochondrial dynamics and integrity drives divergent glycolytic adaptations in podocytes. (A, B) Extracellular acidification rate (ECAR) of *Oma1* ^del^ podocytes measured by Seahorse assays. Data are presented as mean ± SEM for graphs and summary‐plots. *p < 0.05; **p < 0.01; ***p < 0.001; ****p < 0.0001. Wildtype *n* = 18, *Oma1* ^del^ *n* = 23. (C) Intermediates of glycolysis measured in metabolomic studies. Data are presented as log2FC versus control ± SEM. Statistical significance was determined using Student's *t*‐test. *p < 0.05; **p < 0.01; ***p < 0.001; ****p < 0.0001. *n* = 4 for all experimental groups. G6P, glucose‐6‐phosphate; F6P, fructose‐6‐phosphate; FBP, fructose 1,6‐bisphosphate; PEPA, phosphoenolpyruvic acid; PA, pyruvic acid; LA, lactic acid (D) Violin plots of enzymes of glycolysis measured in proteomic studies (E) Expression of enzymes of glycolysis in proteomic studies (log2 fold change versus controls). *n* ≥ 3 for all experimental groups. *FDR < 0.05. ALDOA, aldolase a; ENO1, enolase; GAPDH, glyceraldehyde‐3‐phosphate dehydrogenase; GPI, glucose‐6‐phosphate isomerase; HK1/2, hexokinase 1/2; LDHA/B, lactate dehydrogenase a/b; PCK2, phosphoenolpyruvate carboxykinase 2 (mitochondrial); PFKLM/P, phosphofructokinase (liver/muscle/platelet); PGAM1, phosphoglycerate mutase 1; PGK1, phosphoglycerate kinase; PKM, pyruvate kinase M1/2; TPI1, triosephosphate isomerase 1 (F, G) Extracellular acidification rate (ECAR) of *Phb2* ^kd^ podocytes measured by Seahorse assays. Data are presented as mean ± SEM for graphs and summary‐plots. Statistical significance was determined using Student's *t*‐test. *p < 0.05; **p < 0.01; ***p < 0.001; ****p < 0.0001. shRNA ctrl *n* = 12, *Phb2* ^kd^ *n* = 14.

To investigate the impact of disrupted mitochondrial dynamics and integrity, we utilized Oma1^del^ and Phb2^kd^ podocytes (Figure S1A,B). Successful deletion of OMA1 and efficient knockdown of PHB2 were validated by Western Blot analysis (Figure S1A,B). Transmission electron microscopy (TEM) was used to assess ultrastructure. *Phb2* ^kd^ podocytes exhibited disrupted inner mitochondrial membrane integrity and disorganized cristae, confirmed via quantitative analysis of cristae morphology (Figure S1C,D). *Oma1* ^del^ podocytes showed intact cristae architecture in TEM studies (Figure S1E,F). *Oma1* ^del^ podocytes exhibited significantly enhanced glycolysis in Seahorse studies (Figure 1A,B). This was accompanied by elevated levels of the key glycolytic intermediate fructose 1,6‐bisphosphate (Figure 1C). Proteomic studies further revealed upregulation of multiple glycolytic enzymes, including aldolase A (ALDOA) and the rate‐limiting enzymes hexokinase 2 (HK2) and phosphofructokinase (PFKP, PFKL) (Figure 1D,E, Table S1). Inhibition of mTORC1 through rapamycin treatment (effectiveness confirmed by loss of RPS6 phosphorylation, Figure S2E) not only maintained enhanced glycolysis observed at baseline but also significantly increased the glycolytic capacity of Oma1^del^ podocytes, while glycolytic reserve remained decreased, albeit less in comparison to baseline results (Figure S2A). Notably, stable U‐^13^C_6_ glucose isotope tracer studies of *Oma1* ^del^ podocytes did not show significant differences in ^13^C carbon enrichment in intermediates of glycolysis compared to controls (Figure S5). This suggests that while the overall glycolytic capacity is increased, the turnover rate of these intermediates is rapid enough to maintain steady‐state labeling patterns at this time point. By contrast, *Phb2* ^kd^ podocytes showed no significant change in glycolysis, glycolytic capacity, or glycolytic reserve in Seahorse studies (Figure 1F,G). The metabolome revealed increased levels of fructose 1,6‐bisphosphate but decreased levels of pyruvic acid (Figure 1C). Stable U‐^13^C_6_ glucose isotope tracer studies of *Phb2* ^kd^ podocytes showed no significant alterations in ^13^C carbon enrichment of intermediates of glycolysis versus control at baseline (Figure S5). Furthermore, we observed a decreased expression of the key glycolytic enzyme PFKP in proteomic studies of *Phb2* ^kd^ podocytes (Figure 1D,E, Table S1). This pattern, accumulation of upstream intermediates alongside reduced downstream metabolites and unchanged flux, suggests a metabolic bottleneck rather than enhanced glycolytic throughput. Taken together, *Oma1* ^del^ podocytes robustly enhance glycolytic pathway utilization and enzyme expression, with further amplification by mTORC1 inhibition, while knockdown of PHB2 does not stimulate glycolysis and is associated with reduced expression of critical glycolytic enzymes. Findings of baseline metabolomic and stable isotope tracer studies are summarized in Figure 6A.

### 3.2 Distinct Models of Mitochondrial Dysfunction Reveal Divergent TCA‐Cycle Adaptations and Respiratory Capacity in Podocytes

**FIGURE 2:**
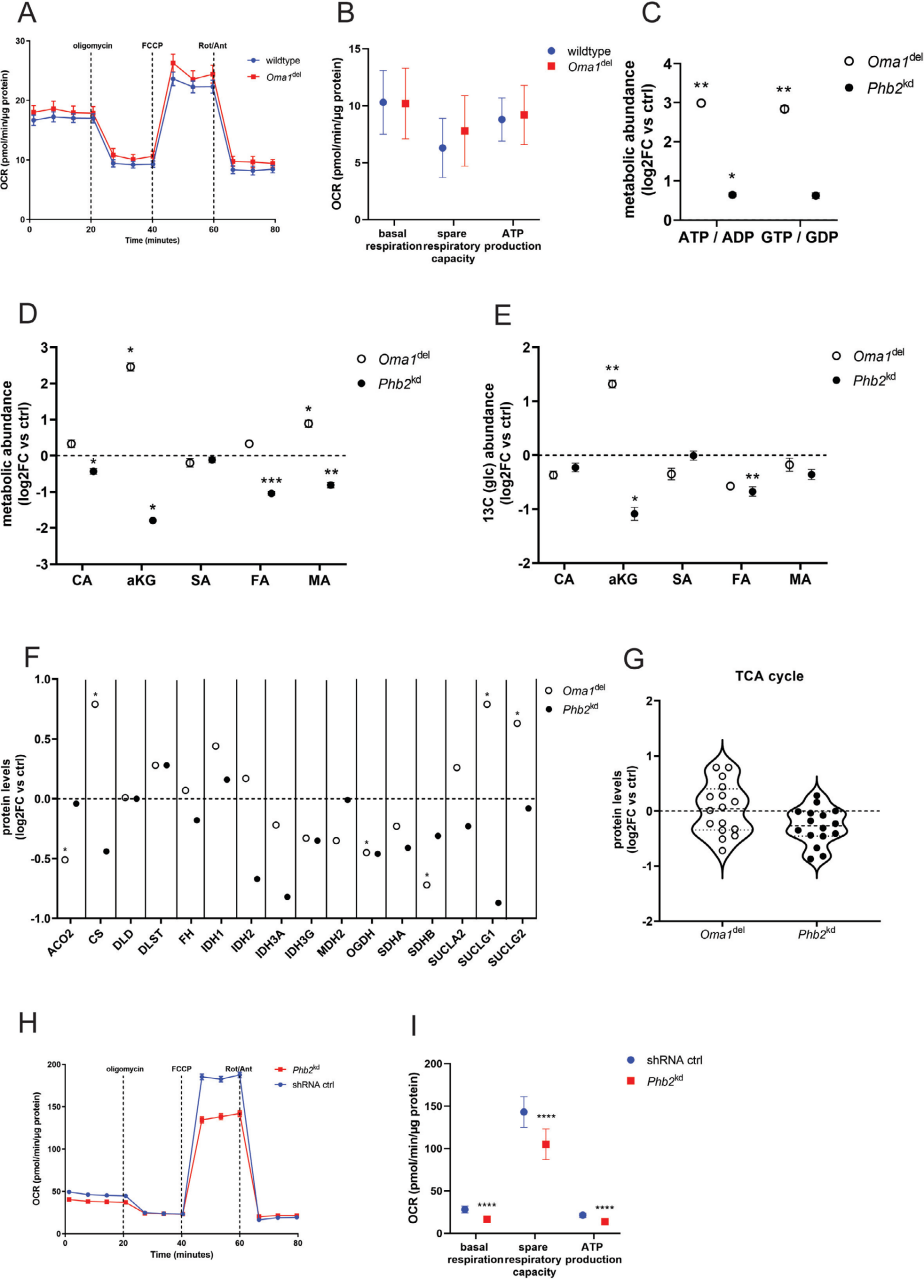
Distinct models of mitochondrial dysfunction reveal divergent TCA‐cycle adaptations and respiratory capacity in podocytes. (A, B) oxidative consumption rate (OCR) of *Oma1* ^del^ podocytes measured by Seahorse assays. Data are presented as mean ± SEM for graphs and summary‐plots. Statistical significance was determined using Student's *t*‐test. *p < 0.05; **p < 0.01; ***p < 0.001; ****p < 0.0001. Wildtype *n* = 40, *Oma*1^del^ *n* = 35. (C) ATP/ADP and GTP/GDP ratios measured in metabolomic studies. Data are presented as mean log2FC versus control ± SEM. Statistical significance was determined using Student's *t*‐test. *p < 0.05; **p < 0.01; ***p < 0.001; ****p < 0.0001, *n* = 4 for all experimental groups. (D) TCA‐cycle intermediates measured in metabolomic studies. Data are presented as log2FC versus control ± SEM. Statistical significance was determined using Student's *t*‐test. *p < 0.05; **p < 0.01; ***p < 0.001; ****p < 0.0001. *n* = 4 for all experimental groups. CA, citric acid; aKG, α‐ketoglutarate; SA, succinic acid; FA, fumaric acid; MA, malic acid. (E) Stable isotope enrichment studies after treatment of cells with U‐^13^C_6_ glucose for 120 min. Data are presented as log2FC versus control ± SEM. Statistical significance was determined using Student's *t*‐test. *p < 0.05; **p < 0.01; ***p < 0.001; ****p < 0.0001. *n* = 4 for all experimental groups. CA, citric acid; aKG, α‐ketoglutarate; SA, succinic acid; FA, fumaric acid; MA, malic acid (F) Expression of TCA cycle enzymes in proteomic studies (log2 fold change versus controls). *n* ≥ 3 for all experimental groups. *FDR < 0.05. ACO2, aconitase 2; CS, citrate synthase; DLD, dihydrolipoamide dehydrogenase; DLST, dihydrolipoamide s‐succinyltransferase; FH, fumarate hydratase; IDH1, cytoplasmic isocitrate dehydrogenase [NADP]; IDH2, mitochondrial isocitrate dehydrogenase [NADP]; IDH3A, isocitrate dehydrogenase (NAD(+)) 3 catalytic subunit alpha; IDH3G, isocitrate dehydrogenase (NAD(+)) 3 non‐catalytic subunit gamma; MDH2, malate dehydrogenase 2; OGDH, oxoglutarate dehydrogenase; SDHA, succinate dehydrogenase complex flavoprotein subunit a; SDHB, succinate dehydrogenase complex iron sulfur subunit b; SUCLA2, succinate‐CoA ligase ADP‐forming subunit beta; SUCLG1, succinate‐CoA ligase GDP/ADP‐forming subunit alpha; SUCLG2, succinate‐CoA ligase GDP/ADP‐forming subunit beta (G) Violin plots of TCA cycle enzymes measured in proteomic studies (H, I) oxidative consumption rate (OCR) of *Phb2* ^kd^ podocytes measured by Seahorse assays. Data are presented as mean ± SEM for graphs and summary‐plots. Statistical significance was determined using Student's t‐test. *p < 0.05; **p < 0.01; ***p < 0.001; ****p < 0.0001. shRNA ctrl *n* = 39, *Phb2* ^kd^ *n* = 37.

*Oma1* ^del^ podocytes showed no significant changes in basal respiration, spare respiratory capacity, or ATP production in Seahorse studies (Figure 2A,B). Semi‐targeted metabolite analysis showed increased amounts of TCA cycle intermediates alpha‐ketoglutarate and malic acid in *Oma1* ^del^ podocytes, with ATP/ADP and GTP/GDP ratios significantly increased versus controls (Figure 2C,D). In stable U‐^13^C_6_ glucose isotope tracer studies, we observed increased ^13^C carbon enrichment in alpha‐ketoglutarate (Figure 2E). Proteome analysis revealed an increased expression of key TCA‐cycle proteins citrate synthase (CS) and Succinyl‐CoA ligase (SUCLG1, 2), while the expression of aconitase 2 (ACO2), succinate dehydrogenase (SDHB), and oxoglutarate dehydrogenase (OGDH) was decreased (Figure 2F,G, Table S1). *Phb2* ^kd^ podocytes showed decreased basal respiration, spare respiratory capacity, and ATP production in Seahorse studies (Figure 2H,I). Interestingly, the ATP/ADP ratio in *Phb2* ^kd^ podocytes was slightly increased at baseline (Figure 2C). This paradoxical increase, despite reduced respiratory function, suggests a significant decrease in cellular energy consumption or a hypometabolic state. After mTORC1 inhibition with rapamycin, *Phb2* ^kd^ podocytes conversely showed an increase in spare respiratory capacity (Figure S2B). Additionally, we observed a significant decrease in the TCA‐cycle intermediates citric acid, alpha‐ketoglutarate, fumaric acid, and malic acid in *Phb2* ^kd^ podocytes at baseline (Figure 2D). Stable U‐^13^C_6_ glucose isotope tracer studies of *Phb2* ^kd^ podocytes revealed decreased ^13^C carbon enrichment in alpha‐ketoglutarate and fumaric acid (Figure 2E). Moreover, we observed a trend of decreased expression of key TCA‐cycle proteins CS, isocitrate dehydrogenase 2 (IDH2, 3a), and SUCLG1 in proteomic studies, which did not reach statistical significance (FDR < 0.05) (Figure 2F,G, Table S1). Taken together, *Oma1* ^del^ podocytes exhibited remodeling and maintenance of TCA cycle function, while the knockdown of PHB2 led to diminished TCA cycle utilization, impaired respiratory capacity, and decreased TCA cycle enzyme expression. Findings of baseline metabolomic and stable isotope tracer studies are summarized in Figure 6A.

### 3.3 Oma1 Deficiency Enhances Glutamine Utilization, While Loss of Phb2 Disrupts Glutamine Anaplerosis

**FIGURE 3:**
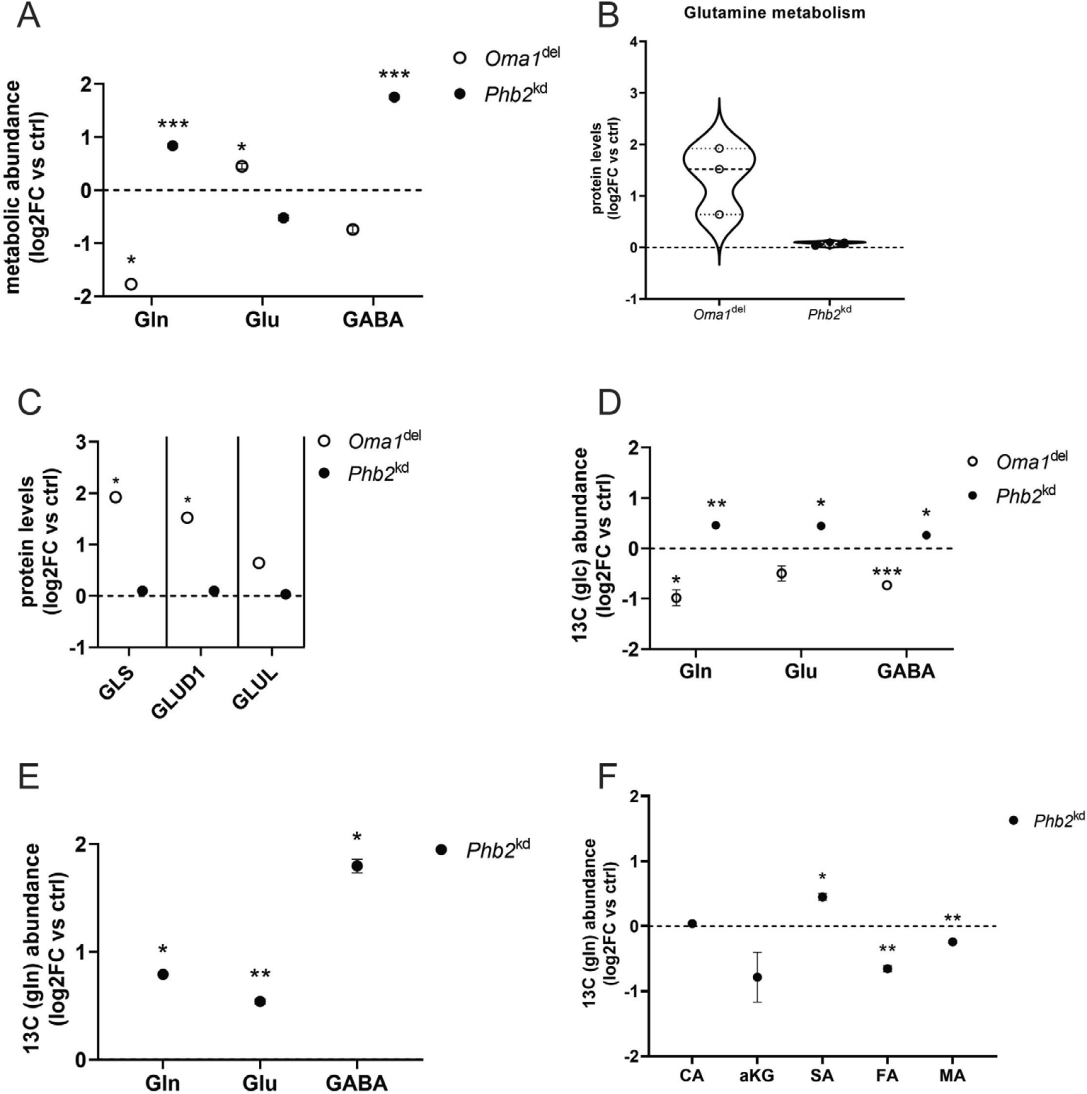
Oma1 deficiency enhances glutamine utilization, while loss of Phb2 disrupts glutamine anaplerosis. (A) Intermediates of glutamine metabolism measured in metabolomic studies. Data are presented as log2FC versus control ± SEM. Statistical significance was determined using Student's t‐test. *p < 0.05; **p < 0.01; ***p < 0.001; ****p < 0.0001. *n* = 4 for all experimental groups. Gln, glutamine; Glu, glutamic acid; GABA, gamma‐aminobutyric acid (B) Violin plots of enzymes of glutamine metabolism measured in proteomic studies (C) Expression of enzymes of glutamine metabolism in proteomic studies (log2 fold change versus controls). *n* ≥ 3 for all experimental groups. *FDR < 0.05. GLS, glutaminase; GLUL, glutamine synthetase; GLUD1, glutamate dehydrogenase 1 (D) Stable isotope enrichment studies after treatment of cells with U‐^13^C_6_ glucose for 120 min. Data are presented as log2FC versus control ± SEM. Statistical significance was determined using Student's *t*‐test. *p < 0.05; **p < 0.01; ***p < 0.001; ****p < 0.0001. *n* = 4 for all experimental groups. Gln, glutamine; Glu, glutamic acid; GABA, gamma‐aminobutyric acid. (E) Stable isotope enrichment studies after treatment of cells with U‐^13^C_5_ glutamine for 120 min. Data are presented as log2FC versus control ± SEM. Statistical significance was determined using Student's *t*‐test. *p < 0.05; **p < 0.01; ***p < 0.001; ****p < 0.0001. *n* ≥ 3 for all experimental groups. Gln, glutamine; Glu, glutamic acid; GABA, gamma‐aminobutyric acid (F) Stable isotope enrichment studies after treatment of cells with U‐^13^C_5_ glutamine for 120 min. Data are presented as log2FC versus control ± SEM. Statistical significance was determined using Student's *t*‐test. *p < 0.05; **p < 0.01; ***p < 0.001; ****p < 0.0001. *n* ≥ 3 for all experimental groups. CA, citric acid; aKG, alpha‐ketoglutarate; SA, succinic acid; FA, fumaric acid; MA, malic acid; GABA, gamma‐aminobutyric acid.

*Oma1* ^del^ podocytes showed increased levels of glutamic acid, with glutamine concurrently decreased in metabolomic studies (Figure 3A). The proteome revealed significantly increased expression of key proteins of glutamine metabolism, glutaminase (GLS) and glutamate dehydrogenase 1 (GLUD1) (Figure 3B,C, Table S1). Stable U‐^13^C_6_ glucose isotope tracer studies of *Oma1* ^del^ podocytes showed decreased ^13^C carbon enrichment in glutamine and GABA (Figure 3D). *Phb2* ^kd^ podocytes showed increased levels of glutamine and GABA in metabolomic studies (Figure 3A). Stable U‐^13^C_6_ glucose isotope tracer studies of *Phb2* ^kd^ podocytes showed increased ^13^C carbon enrichment in glutamine, glutamic acid, and GABA (Figure 3D). To further assess glutamine metabolism in *Phb2* ^kd^ podocytes, we conducted stable U‐^13^C_5_ glutamine isotope tracer studies, where we observed increased ^13^C carbon enrichment in glutamic acid, GABA, and succinic acid, while ^13^C carbon enrichment was decreased in fumaric acid and malic acid (Figure 3E,F). This pattern indicates a bottleneck in the latter half of the TCA cycle, impairing the complete oxidation of glutamine‐derived carbons. Taken together, *Oma1* ^del^ podocytes showed enhanced glutamine catabolism, whereas loss of PHB2 disrupted glutamine metabolism, leading to an accumulation of glutamine and GABA with impaired glutamine carbon utilization into the TCA cycle. Findings of baseline metabolomic and stable isotope tracer studies are summarized in Figure 6A.

### 3.4 Insulin Treatment Induces Distinct Proteomic Remodeling in Oma1^del^ and Phb2^kd^ Podocytes

**FIGURE 4:**
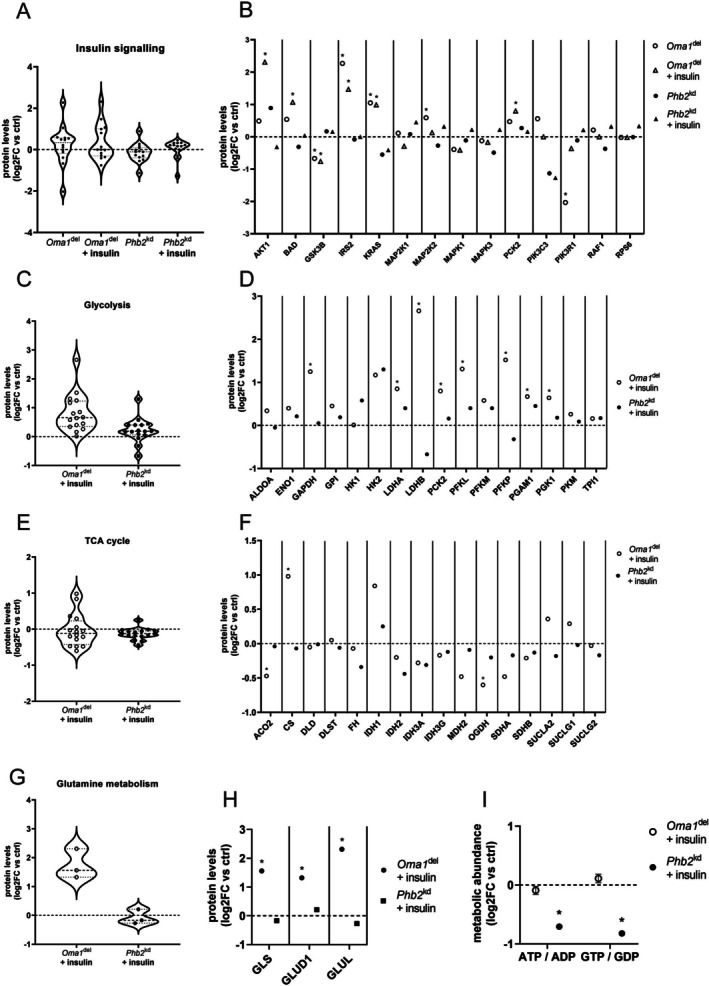
Insulin treatment induces distinct proteomic remodeling in Oma1^del^ and Phb2^kd^ podocytes. (A) Violin plots of insulin signaling proteins measured in proteomic studies before and after insulin‐treatment (10 μg/mL medium over 3 h) (B) Expression of insulin signaling proteins measured in proteomic studies before and after insulin stimulation (10 μg/mL medium over 3 h) (log2 fold change versus controls). *n* ≥ 3 for all experimental groups. *FDR < 0.05. AKT1, RAC‐alpha serine/threonine‐protein kinase; BAD, Bcl2‐associated agonist of cell death; GSK3B, glycogen synthase kinase‐3 beta; IRS2, insulin receptor substrate 2; KRAS, GTPase KRas; MAP2K1/2, dual specificity mitogen‐activated protein kinase kinase 1/2; MAPK3, mitogen‐activated protein kinase 3; PCK2, phosphoenolpyruvate carboxykinase 2 (mitochondrial); PIK3C3, phosphatidylinositol 3‐kinase catalytic subunit type 3; PIK3R1, phosphatidylinositol 3‐kinase regulatory subunit alpha; RAF1, RAF proto‐oncogene serine/threonine‐protein kinase; RPS6, 40S ribosomal protein S6 (C) Violin plots of enzymes of glycolysis measured in proteomic studies after insulin‐treatment (10 μg/mL medium over 3 h) (D) Expression of enzymes of glycolysis measured in proteomic studies after insulin treatment (10 μg/mL medium over 3 h) (log2 fold change versus controls). *n* ≥ 3 for all experimental groups. *FDR < 0.05. ALDOA, aldolase a; ENO1, enolase; GAPDH, glyceraldehyde‐3‐phosphate dehydrogenase; GPI, glucose‐6‐phosphate isomerase; HK1/2, hexokinase 1/2; LDHA/B, lactate dehydrogenase a/b; PCK2, phosphoenolpyruvate carboxykinase 2 (mitochondrial); PFKLM/P, phosphofructokinase (liver/muscle/platelet); PGAM1, phosphoglycerate mutase 1; PGK1, phosphoglycerate kinase; PKM, pyruvate kinase M1/2; TPI1, triosephosphate isomerase 1 (E) Violin plots of TCA cycle enzymes measured in proteomic studies after insulin‐treatment (10 μg/mL medium over 3 h) (F) Expression of TCA cycle enzymes in proteomic studies after insulin treatment (10 μg/mL medium over 3 h) (log2 fold change versus controls). *n* ≥ 3 for all experimental groups. *FDR < 0.05. ACO2, aconitase 2; CS, citrate synthase; DLD, dihydrolipoamide dehydrogenase; DLST, dihydrolipoamide s‐succinyltransferase; FH, fumarate hydratase; IDH1, cytoplasmic isocitrate dehydrogenase [NADP]; IDH2, mitochondrial isocitrate dehydrogenase [NADP]; IDH3A, isocitrate dehydrogenase (NAD(+)) 3 catalytic subunit alpha; IDH3G, isocitrate dehydrogenase (NAD(+)) 3 non‐catalytic subunit gamma; MDH2, malate dehydrogenase 2; OGDH, oxoglutarate dehydrogenase; SDHA, succinate dehydrogenase complex flavoprotein subunit a; SDHB, succinate dehydrogenase complex iron sulfur subunit b; SUCLA2, succinate‐CoA ligase ADP‐forming subunit beta; SUCLG1, succinate‐CoA ligase GDP/ADP‐forming subunit alpha; SUCLG2, succinate‐CoA ligase GDP/ADP‐forming subunit beta (G) Violin plots of enzymes of glutamine metabolism measured in proteomic studies after insulin‐treatment (10 μg/mL medium over 3 h) (H) Expression of enzymes of glutamine metabolism in proteomic studies after insulin treatment (10 μg/mL medium over 3 h) (log2 fold change versus controls). *n* ≥ 3 for all experimental groups. *FDR < 0.05. GLS, glutaminase; GLUL, glutamine synthetase; GLUD1, glutamate dehydrogenase 1 (I) ATP/ADP and GTP/GDP ratios measured in metabolomic studies after insulin treatment (10 μg/mL medium over 3 h). Data are presented as log2FC versus control ± SEM. Statistical significance was determined using Student's *t*‐test. *p < 0.05; **p < 0.01; ***p < 0.001; ****p < 0.0001. *n* = 3 for all experimental groups.

To deepen our understanding of how these distinct mitochondrial disruptions impact metabolic signaling in podocytes, we assessed bioenergetic, proteomic, and metabolic profiles of *Oma1* ^del^ and *Phb2* ^kd^ podocytes after treatment with insulin. At baseline, we observed increased expression of insulin signaling proteins insulin receptor substrate 2 (IRS2), KRAS proto‐oncogene (KRAS), and mitogen‐activated protein kinase kinase 2 (MAP2K2) in *Oma1* ^del^ podocytes, while expression of glycogen synthase kinase 3 beta (GSK3B) and phosphoinositide‐3‐kinase regulatory subunit 1 (PIK3R1) was decreased (Figure 4A,B, Table S1). After insulin treatment of *Oma1* ^del^ podocytes, we saw an increased expression of AKT1, BAD, IRS2, KRAS, and phosphoenolpyruvate carboxykinase 2 (PCK2), while expression of GSK3B continued to be decreased (Figure 4A,B, Table S1). Furthermore, we observed an increased expression of glycolytic enzymes GAPDH, LDHA, LDHB, PCK2, PFKL, PFKP, phosphoglycerate mutase (PGAM1), and phosphoglycerate kinase 1 (PGK1) (Figure 4C,D, Table S1). Moreover, we saw increased expression of TCA‐cycle protein CS, while TCA cycle proteins ACO2 and OGDH were significantly less expressed (Figure 4E,F, Table S1). Furthermore, *Oma1* ^del^ podocytes continued to exhibit increased expression of the key proteins of glutamine metabolism GLS, GLUD1, and glutamate‐ammonia ligase (GLUL) (Figure 4G,H, Table S1). In *Phb2* ^kd^ podocytes, we observed no increased expression of insulin signaling proteins at baseline, but decreased expression of mitogen‐activated protein kinase 3 (MAPK3), PIK3C3, and KRAS (Figure 4A,B, Table S1). After treatment with insulin, only PIK3C3 expression remained decreased in *Phb2* ^kd^ podocytes, highlighting an overall attenuated insulin response (Figure 4A,B, Table S1). In proteomic studies, *Phb2* ^kd^ podocytes treated with insulin showed an increased expression of HK2, while expression of LDHB was decreased (Figure 4C,D, Table S1). Taken together, while insulin treatment induced an upregulation of key signaling and metabolic protein networks in Oma1^del^ podocytes, Phb2^kd^ podocytes were largely unresponsive, exhibiting a failure for proteomic adaptation. Findings of metabolomic and stable isotope tracer studies after insulin treatment are summarized in Figure 6B.

### 3.5 Oma1 Deficiency Enhances Metabolic Flexibility Upon Insulin Stimulation, While Phb2 Knockdown Leads to Metabolic Failure

**FIGURE 5:**
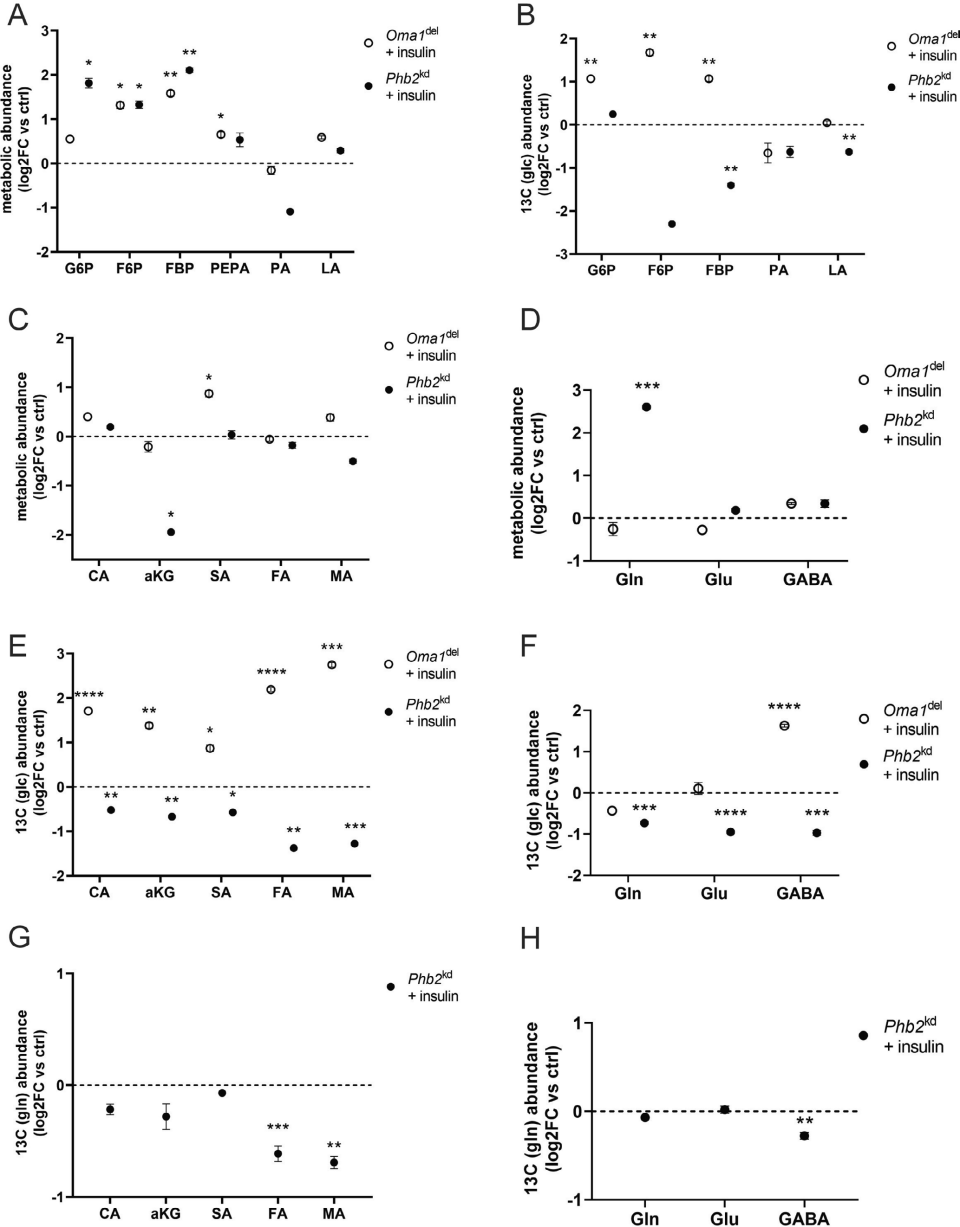
Oma1 deficiency enhances metabolic flexibility upon insulin stimulation, while Phb2 knockdown leads to metabolic failure. (A) Metabolites measured in metabolomic studies after insulin treatment (10 μg/mL medium over 3 h). Data are presented as log2FC versus control ± SEM. Statistical significance was determined using Student's t‐test. *p < 0.05; **p < 0.01; ***p < 0.001; ****p < 0.0001. *n* = 3 for all experimental groups. G6P: glucose‐6‐phosphate, F6P: fructose‐6‐phosphate, FBP: fructose‐1,6‐bisphosphate, PEPA: phosphoenolpyruvic acid, PA: pyruvic acid, LA: lactic acid. (B) Stable isotope enrichment studies after treatment of insulin‐treated cells (10 μg/mL medium over 3 h) with U‐^13^C_6_ glucose for 120 min. Data are presented as log2FC versus control ± SEM. Statistical significance was determined using Student's *t*‐test. *p < 0.05; **p < 0.01; ***p < 0.001; ****p < 0.0001. *n* = 4 for all experimental groups. G6P, glucose‐6‐phosphate; F6P, fructose‐6‐phosphate; FBP, fructose‐1,6‐bisphosphate; PA, pyruvic acid; LA, lactic acid. (C) Metabolites measured in metabolomic studies after insulin treatment (10 μg/mL medium over 3 h). Data are presented as log2FC versus control ± SEM. Statistical significance was determined using Student's *t*‐test. *p < 0.05; **p < 0.01; ***p < 0.001; ****p < 0.0001. *n* = 3 for all experimental groups. CA, citric acid; aKG, alpha‐ketoglutarate; SA, succinic acid; FA, fumaric acid; MA, malic acid. (D) Metabolites measured in metabolomic studies after insulin treatment (10 μg/mL medium over 3 h). Data are presented as log2FC versus control ± SEM. Statistical significance was determined using Student's t‐test. *p < 0.05; **p < 0.01; ***p < 0.001; ****p < 0.0001. *n* = 3 for all experimental groups. Gln, glutamine; Glu, glutamic acid; GABA, gamma‐aminobutyric acid (E) Stable isotope enrichment studies after treatment of insulin‐treated cells (10 μg/mL medium over 3 h) with U‐^13^C_6_ glucose for 120 min. Data are presented as log2FC versus control ± SEM. Statistical significance was determined using Student's *t*‐test. *p < 0.05; **p < 0.01; ***p < 0.001; ****p < 0.0001. *n* = 4 for all experimental groups. CA, citric acid; aKG, alpha‐ketoglutarate; SA, succinic acid; FA, fumaric acid; MA, malic acid. (F) Stable isotope enrichment studies after treatment of insulin‐treated cells (10 μg/mL medium over 3 h) with U‐^13^C_6_ glucose for 120 min. Data are presented as log2FC versus control ± SEM. Statistical significance was determined using Student's *t*‐test. *p < 0.05; **p < 0.01; ***p < 0.001; ****p < 0.0001. *n* = 4 for all experimental groups. Gln, glutamine; Glu, glutamic acid; GABA, gamma‐aminobutyric acid. (G) Stable isotope enrichment studies after treatment of insulin‐treated cells (10 μg/mL medium over 3 h) with U‐^13^C_5_ glutamine for 120 min. Data are presented as log2FC versus control ± SEM. Statistical significance was determined using Student's *t*‐test. *p < 0.05; **p < 0.01; ***p < 0.001; ****p < 0.0001. *n* ≥ 3 for all experimental groups. CA, citric acid; aKG, alpha‐ketoglutarate; SA, succinic acid; FA, fumaric acid; MA, malic acid. (H) Stable isotope enrichment studies after treatment of insulin‐treated cells (10 μg/mL medium over 3 h) with U‐^13^C_5_ glutamine for 120 min. Data are presented as log2FC versus control ± SEM. Statistical significance was determined using Student's *t*‐test. *p < 0.05; **p < 0.01; ***p < 0.001; ****p < 0.0001. *n* ≥ 3 for all experimental groups. Gln, glutamine; Glu, glutamic acid; GABA, gamma‐aminobutyric acid.

**FIGURE 6:**
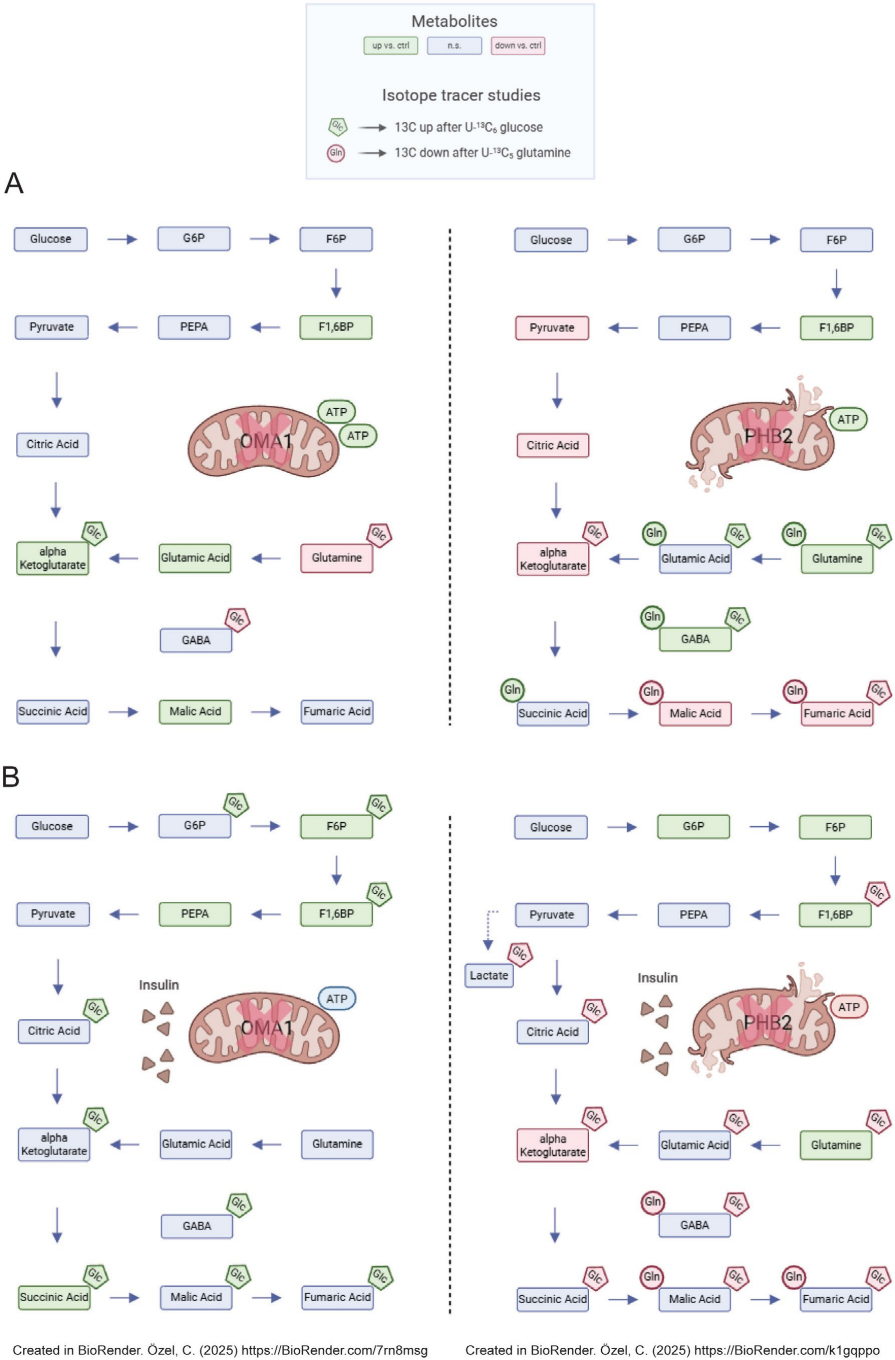
Summary of results of metabolomic and stable isotope tracing studies. Schematic of results of metabolomic and stable isotope tracing studies at baseline (A) and following insulin treatment (B). Left: *Oma1* ^del^ podocytes, right: *Phb2* ^kd^ podocytes. Blue: Not significantly altered versus control. Green: Significantly enhanced versus control. Red: Significantly reduced versus control. Glc: 13C glucose enrichment in U‐^13^C_6_ isotope tracer studies. Gln: 13C glutamine enrichment in ^13^C_5_ glutamine isotope tracer studies. (A) *Oma1* ^del^ podocytes exhibit increased abundance of fructose‐1,6‐bisphosphate, alpha‐ketoglutarate, glutamic acid, malic acid and high levels of ATP while glutamine levels are decreased versus controls. In tracer studies after treatment with U‐^13^C_6_ glucose, there is increased 13C enrichment in alpha‐ketoglutarate and decreased enrichment in Glutamine and GABA. *Phb2* ^kd^ podocytes show increased levels of fructose‐1,6‐bisphosphate, Glutamine, GABA and ATP, while levels of pyruvate, citric acid, alpha‐ketoglutarate, malic acid, and fumaric acid were decreased. In tracer studies after treatment with U‐^13^C_6_ glucose, there is increased 13C enrichment in Glutamine, Glutamic acid and GABA, while 13C enrichment was decreased in alpha‐ketoglutarate and fumaric acid. In tracer studies after treatment with U‐^13^C_5_ glutamine, there is increased 13C enrichment in Glutamic acid, GABA and succinic acid, while 13C enrichment was decreased in malic and fumaric acid. (B) Following treatment with insulin, *Oma1* ^del^ podocytes exhibit increased abundance of fructose‐6‐phosphate, fructose‐1,6‐bisphosphate, phosphoenolpyruvic acid, and succinic acid. In tracer studies after treatment with U‐^13^C_6_ glucose, there is increased 13C enrichment in glucose‐6‐phosphate, fructose‐6‐phosphate, fructose‐1,6‐bisphosphate, citric acid, alpha‐ketoglutarate, succinic acid, malic acid, fumaric acid, and GABA. *Phb2* ^kd^ podocytes show increased levels of glucose‐6‐phosphate, fructose‐6‐phosphate, fructose‐1,6‐bisphosphate and glutamine, while levels of alpha‐ketoglutarate ant ATP are decreased. In tracer studies after treatment with U‐^13^C_6_ glucose, there is decreased 13C enrichment in fructose‐1,6‐bisphosphate, lactic acid, citric acid, alpha‐ketoglutarate, succinic acid, malic acid, fumaric acid, glutamine, glutamic acid, and GABA. In tracer studies after treatment with U‐^13^C_5_ glutamine, there is decreased 13C enrichment in GABA, malic and fumaric acid.

Treatment of *Oma1* ^del^ podocytes with insulin resulted in increased levels of fructose‐6‐phosphate, fructose 1,6‐bisphosphate, PEPA, and succinic acid (Figure 5A,C). Stable U‐^13^C_6_ glucose isotope tracer studies after insulin treatment of *Oma1* ^del^ podocytes showed increased ^13^C carbon enrichment in glucose‐6‐phosphate, fructose‐6‐phosphate, and fructose 1,6‐bisphosphate (Figure 5B). *Phb2* ^kd^ podocytes responded to insulin treatment with increased levels of glucose‐6‐phosphate, fructose‐6‐phosphate, fructose 1,6‐bisphosphate, and glutamine, while alpha‐ketoglutarate was decreased (Figure 5A,C,D). Stable U‐^13^C_6_ glucose isotope tracer studies after insulin treatment of *Phb2* ^kd^ podocytes showed decreased ^13^C carbon enrichment in citric acid, alpha‐ketoglutarate, succinic acid, fumaric acid, malic acid, fructose 1,6‐bisphosphate, lactic acid, glutamine, glutamic acid, and GABA (Figure 5B,E,F). Stable U‐^13^C_5_ glutamine isotope tracer studies after insulin treatment of *Phb2* ^kd^ podocytes showed decreased ^13^C carbon enrichment in fumaric acid, malic acid, and GABA (Figure 5G,H). Furthermore, we observed a decreased ATP/ADP and GTP/GDP ratio in *Phb2* ^kd^ podocytes treated with insulin, indicating an energetic collapse when metabolically challenged (Figure 5I). Taken together, insulin promotes glycolytic flux in Oma1^del^ podocytes, but triggers a failure of central carbon metabolism in Phb2^kd^ podocytes, with an inability to funnel both glucose and glutamine into the TCA cycle and consecutive bioenergetic collapse. Findings of metabolomic and stable isotope tracer studies after insulin treatment are summarized in Figure 6B.

## 4 Discussion

In this study, we examined the impact of mitochondrial dysfunction on podocyte metabolism using models that impair mitochondrial dynamics without directly disabling OXPHOS. Specifically, we investigated the role of OPA1, a critical regulator of mitochondrial dynamics, by preventing its cleavage through genetic deletion of *Oma1*, the protease responsible for this process, or knockdown of *Phb2*, a key modulator of OMA1 activity. Our findings highlight that podocytes respond to altered mitochondrial dynamics with distinct metabolic reprogramming.

In essence, preserving mitochondrial fusion via loss of OMA1 triggers a compensatory shift towards glycolysis and anaplerosis, whereas disrupting inner membrane integrity via loss of PHB2 leads to a profound metabolic collapse. This observation seemingly conflicts with the simple notion that increased mitochondrial fusion supports efficient OXPHOS and excessive fission is associated with a compensatory increase in glycolysis [38]. However, the divergent outcomes likely stem from the distinct structural roles of these proteins beyond regulating morphology. PHB2 is essential for maintaining the architecture of the inner mitochondrial membrane (IMM) and cristae junctions [39]. This architecture facilitates the organization of respiratory chain supercomplexes and metabolite transporters into functional units, which enable efficient substrate channeling [40]. The metabolic failure in Phb2kd podocytes is likely a direct consequence of disrupted IMM integrity and cristae organization. This structural collapse likely impairs substrate channeling and the organization of respiratory supercomplexes, severely impairing metabolite flux, independent of the observed excessive fission. This underscores that the structural integrity of the IMM is the prerequisite for metabolic competence in podocytes.

The consequences of this structural disruption are evident in the failure of Phb2^kd^ podocytes to maintain anaplerotic competence. Despite elevated cellular glutamine levels, reduced alpha‐ketoglutarate and diminished 13C incorporation into TCA cycle intermediates indicate severe anaplerotic insufficiency, constraining TCA cycle flux. Glutamine anaplerosis is essential for maintaining TCA cycle function when pyruvate supply to mitochondria is limited [41]. A reduced capacity of glutamine‐derived anaplerotic feeding of the TCA cycle can be observed in rodent studies resembling human inherited metabolic disorders, highlighting glutamine anaplerosis as a potential therapeutic intervention point in methylmalonic aciduria [42]. A key distinction between the models lies in the interpretation of elevated fructose‐1,6‐bisphosphate levels observed in both genotypes. In Oma1^del^ podocytes, this elevation aligns with enhanced extracellular acidification rate and upregulation of rate‐limiting enzymes, indicating increased glycolytic flux. In contrast, Phb2^kd^ podocytes exhibit elevated fructose‐1,6‐bisphosphate despite unchanged ECAR and decreased pyruvate levels. This suggests that in Phb2^kd^ cells, the accumulation of upstream intermediates results from a downstream metabolic blockade rather than enhanced throughput. This interpretation is further supported by the insulin response data, where Phb2^kd^ podocytes show increased G6P/FBP levels concurrent with reduced 13C enrichment. This uncoupling suggests pathological accumulation due to the inability to utilize carbon substrates downstream. Interestingly, Phb2^kd^ cells exhibited a slightly increased ATP/ADP ratio at baseline despite reduced respiration. This paradox indicates a profound reduction in ATP turnover. It is important to note that these experiments were conducted in differentiated podocytes cultured at 37°C for 10 days. Under these conditions, podocytes are known to exit the cell cycle and adopt a post‐mitotic, quiescent state [43]. Therefore, the observed reduction in ATP consumption cannot be attributed to differences in proliferation rates. Instead, it suggests the cells enter a hypometabolic state, curtailing other energy‐intensive cellular processes to conserve ATP [44]. However, this adaptive strategy masks an underlying vulnerability. Upon insulin stimulation, the energetic state (ATP/ADP and GTP/GDP ratios) collapses, highlighting their metabolic fragility.

Conversely, Oma1^del^ podocytes demonstrated metabolic resilience characterized by anaplerotic competence. They exhibited upregulation of glutamine‐utilizing enzymes and increased alpha‐ketoglutarate, indicating efficient fueling of the TCA cycle. The upregulated citrate synthase alongside downregulated aconitase 2 and oxoglutarate dehydrogenase suggests a remodeling of TCA cycle flux. This configuration may favor reductive carboxylation, where the TCA cycle runs in reverse, utilizing glutamine‐derived carbons for biosynthesis and maintaining redox balance rather than complete oxidation [45]. *Oma1* ^del^ podocytes showed an upregulation of glutamine‐utilizing enzymes, decreased levels of glutamine, and increased levels of alpha‐ketoglutarate, suggesting that glutamine is converted to aKG to replenish TCA cycle intermediates and sustain energetic demands. Following insulin treatment, however, ^13^C_6_ glucose tracer studies revealed an increased incorporation of labeled carbons into GABA and succinic acid (SA), indicating that *Oma1* ^del^ podocytes respond to insulin stimulation by activating the GABA shunt. The GABA shunt bypasses portions of the TCA cycle by converting glutamate to GABA and then into succinate, which re‐enters the TCA cycle to maintain its function when certain steps are saturated or slowed [46]. This bypass mechanism may be crucial for maintaining anaplerosis and redox balance when the conventional TCA cycle flow is constrained by the observed downregulation of ACO2 and OGDH. Collectively, our findings indicate that *Oma1* ^del^ podocytes utilize glutamine metabolism and alternative routing to maintain TCA cycle function both at baseline and after treatment with insulin. Interestingly, *Oma1* ^del^ podocytes exhibited elevated ATP/ADP and GTP/GDP ratios at baseline that diminished after treatment with insulin, suggesting an inability to respond to insulin with a further increase of glycolysis, as it might already be near full capacity at baseline.

In a colorectal cancer model, OMA1 promoted a shift towards glycolysis via HIF‐1α stabilization, effectively suppressing OXPHOS in favor of glycolytic ATP production [47]. This is further supported by a recent study of cells with DNA damage, where OMA1‐deficient cells showed reduced glycolysis that could be restored by OXPHOS inhibition [48]. However, in our studies, we observed a shift towards glycolysis in podocytes with genetic deletion of *Oma1*, suggesting cell type‐specific metabolic adaptations to mitochondrial dysfunction. In podocytes of diabetic mice, enhancing glycolysis through overexpression of pyruvate kinase M2 (PKM2) has been shown to prevent kidney injury and restore mitochondrial function, highlighting the renoprotective effects of increased glycolysis when mitochondrial function is compromised [49]. Furthermore, in rodent studies, mice with neuron‐specific PHB2 deficiency exhibited neurodegenerative disease with a reduced lifespan that could be rescued by additional ablation of *Oma1* [50]. Likewise, in acute kidney injury models, OMA1 activation was shown to exacerbate kidney injury, and inhibition of OMA1 rescued renal function and protected proximal tubular cells from apoptosis [51]. In a rodent diabetic nephropathy model, treatment with the mitochondria‐targeted peptide SS31 reduced OMA1 levels, restored mitochondrial function, and mitigated podocyte injury [52]. *Oma1* ^del^ podocytes might be protected from injury by increased glycolysis and utilization of alternative metabolic pathways, whereas *Phb2* ^kd^ podocytes might be more vulnerable because of a reduced capacity for anaplerotic feeding of the TCA cycle and a reduced ability to perform a metabolic shift to survive stress. However, chronic reliance on glycolysis might have deleterious byproducts (e.g., lactate accumulation, NAD^+^ depletion), and not all aspects of mitochondrial function might be rescued by dynamic adjustment of cellular metabolism. Indeed, whole‐body deletion of Oma1 has been linked to obesity, reduced thermogenesis, and aggravation of cardiomyopathy in rodent studies [53, 54]. Mitochondrial stress responses, like OMA1 activation, might be helpful for adapting metabolism in the short term but potentially harmful if the primary metabolic defect (e.g., anaplerotic failure) is not resolved.

Several limitations must be acknowledged. A key consideration is the distinct origin of the cell models. Oma1^del^ podocytes were derived from primary mouse kidney isolates, while Phb2^kd^ podocytes were generated in an established immortalized podocyte line. Differences in background or clonal selection may contribute to the observed divergent metabolic phenotypes. Furthermore, our assessment of insulin signaling relied on total protein expression, and we did not measure acute activation status, which may not fully capture the dynamics of the signaling response. While our stable isotope tracing provides insights into substrate utilization, it primarily reflects steady‐state metabolism. Dynamic flux analysis would be required to precisely quantify the rates of these metabolic shifts. Our podocyte models illuminate mechanisms under controlled in vitro conditions; real‐world disease states likely involve additional layers of complexity. For instance, inflammatory signals, substrate availability in vivo, and crosstalk with other cell types could modulate the metabolic outcomes we observed. It remains an open question whether simply enhancing glycolytic capacity or anaplerotic input in podocytes can ameliorate chronic glomerular diseases marked by mitochondrial dysfunction. Our findings show that podocytes respond to changes in mitochondrial morphology with a carefully calibrated metabolic reprogramming to restore energetic balance. Future investigations will need to address whether increasing anaplerosis alongside modulating mitochondrial fission and fusion could synergize to restore podocyte health in glomerular diseases.

## Author Contributions

Cem Özel: Writing – review and editing, writing – original draft, visualization, investigation, formal analysis, and conceptualization. Katrin Reitmeier and Emilia Kiekhöfer: Investigation and formal analysis. Khawla Abualia: Writing – review and editing and formal analysis. Duc Nguyen‐Minh, Mahsa Matin, and Richard J.M. Coward: Writing – review and editing, investigation, and formal analysis. Henning Hagmann and Philipp Antczak: Writing – review and editing and formal analysis. Sebastian Brähler: Writing – review and editing and funding acquisition. Bernhard Schermer: Writing – review and editing, supervision, and conceptualization. Thomas Benzing: Writing – review and editing, supervision, funding acquisition, and conceptualization. Patrick Giavalisco: Writing – review and editing, investigation, formal analysis, and conceptualization. Paul T. Brinkkoetter: Writing – review and editing, supervision, funding acquisition, and conceptualization.

## Funding

This work was supported by the Deutsche Forschungsgemeinschaft as part of the Clinical Research Unit KFO329 and Collaborative Research Center TRR422. PB was supported by research funding from the DFG BR‐2955/8 and from the German Federal Ministry of Education and Research (STOP‐FSGS 01GM1901E). CO was supported by Gerok funds of the Deutsche Forschungsgemeinschaft for a medical research rotation. DNM was supported by the Koeln Fortune Program of the Faculty of Medicine of the University of Cologne. RJC was supported by the Medical Research Council (Senior Clinical Fellowship, MR/K010492/1, MR/W019582/1, MR/T002263/1).

## Conflicts of Interest

The authors declare no conflicts of interest.

## Supporting information


**Table S1:** linked to Figures 1, 2, 3, 4, 5: log2FC and FDR of proteomic studies of enzymes of glycolysis before and after insulin treatment (10 μg/mL medium over 3 h). ALDOA, aldolase a; ENO1, enolase; GAPDH, glyceraldehyde‐3‐phosphate dehydrogenase; GPI, glucose‐6‐phosphate isomerase; HK1/2, hexokinase 1/2; LDHA/B, lactate dehydrogenase a/b; PCK2, phosphoenolpyruvate carboxykinase 2 (mitochondrial); PFKLM/P, phosphofructokinase (liver/muscle/platelet); PGAM1, phosphoglycerate mutase 1; PGK1, phosphoglycerate kinase; PKM, pyruvate kinase M1/2; TPI1, triosephosphate isomerase 1. ACO2, aconitase 2; CS, citrate synthase; DLD, dihydrolipoamide dehydrogenase; DLST, dihydrolipoamide s‐succinyltransferase; FH, fumarate hydratase; IDH1, cytoplasmic isocitrate dehydrogenase [NADP]; IDH2, mitochondrial isocitrate dehydrogenase [NADP]; IDH3A, isocitrate dehydrogenase (NAD(+)) 3 catalytic subunit alpha; IDH3G, isocitrate dehydrogenase (NAD(+)) 3 non‐catalytic subunit gamma; MDH2, malate dehydrogenase 2; OGDH, oxoglutarate dehydrogenase; SDHA, succinate dehydrogenase complex flavoprotein subunit a; SDHB, succinate dehydrogenase complex iron sulfur subunit b; SUCLA2, succinate‐CoA ligase ADP‐forming subunit beta; SUCLG1, succinate‐CoA ligase GDP/ADP‐forming subunit alpha; SUCLG2, succinate‐CoA ligase GDP/ADP‐forming subunit beta; GLS, glutaminase; GLUL, glutamine synthetase; GLUD1, glutamate dehydrogenase 1; AKT1, RAC‐alpha serine/threonine‐protein kinase; BAD, Bcl2‐associated agonist of cell death; GSK3B, glycogen synthase kinase‐3 beta; IRS2, insulin receptor substrate 2; KRAS, GTPase KRas; MAP2K1/2, dual specificity mitogen‐activated protein kinase kinase 1/2; MAPK3, mitogen‐activated protein kinase 3; PCK2, phosphoenolpyruvate carboxykinase 2 (mitochondrial); PIK3C3, phosphatidylinositol 3‐kinase catalytic subunit type 3; PIK3R1, phosphatidylinositol 3‐kinase regulatory subunit alpha; RAF1, RAF proto‐oncogene serine/threonine‐protein kinase; RPS6, 40S ribosomal protein S6.

**Figure S1:** Phb2kd podocytes show disrupted cristae morphology in electron microscopy (A) schematic depiction of the generation of Oma1del and Phb2kd podocytes and insulin stimulation protocol (B) schematic depiction of podocyte differentiation and treatment protocol (C) Representative transmission electron microscopy (TEM) images of mitochondria of Phb2kd podocytes and control podocytes (10.000× magnification, scale bar = 500 nm) (D) cristae area/total mitochondrial area ratio measured in TEM images. Data are presented as mean ± SEM. Statistical significance was determined using Student's *t*‐test. *p < 0.05; **p < 0.01; ***p < 0.001; ****p < 0.0001. *n* = 22 for each experimental group. (E) Representative transmission electron microscopy (TEM) images of mitochondria of Oma1del podocytes and control podocytes (10.000× magnification, scale bar = 500 nm) (F) cristae area/total mitochondrial area ratio measured in TEM images. Data are presented as mean ± SEM. Statistical significance was determined using Student's *t*‐test. *p < 0.05; **p < 0.01; ***p < 0.001; ****p < 0.0001. *n* = 22 for each experimental group.

**Figure S2:** linked to Figures 1 and 2 (A) Extracellular acidification rate (ECAR) of Oma1del podocytes measured by Seahorse assays after rapamycin exposure (10 ng/mL medium for 2 h). Data are presented as mean ± SEM for graphs and summary‐plots. Statistical significance was determined using Student's *t*‐test. *p < 0.05; **p < 0.01; ***p < 0.001; ****p < 0.0001. Wildtype *n* = 20, Oma1del *n* = 20. (B) Oxidative consumption rate (OCR) of Oma1del podocytes measured by Seahorse assays after rapamycin exposure (10 ng/mL medium for 2 h). Data are presented as mean ± SEM for graphs and summary‐plots. Statistical significance was determined using Student's t‐test. *p < 0.05; **p < 0.01; ***p < 0.001; ****p < 0.0001. Wildtype *n* = 21, Oma1del *n* = 18. (C) Extracellular acidification rate (ECAR) of Phb2kd podocytes measured by Seahorse assays after rapamycin exposure (10 ng/mL medium for 2 h). Data are presented as mean ± SEM for graphs and summary‐plots. Statistical significance was determined using Student's *t*‐test. *p < 0.05; **p < 0.01; ***p < 0.001; ****p < 0.0001. shRNA ctrl *n* = 10, Phb2kd *n* = 21. (D) Oxidative consumption rate (OCR) of Phb2kd podocytes measured by Seahorse assays after rapamycin exposure (10 ng/mL medium for 2 h). Data are presented as mean ± SEM for graphs and summary‐plots. Statistical significance was determined using Student's *t*‐test. *p < 0.05; **p < 0.01; ***p < 0.001; ****p < 0.0001. shRNA ctrl *n* = 21, Phb2kd *n* = 20.

**Figure S3:** linked to Figures 1, 2, 3, 4, 5 (A) PCA of proteomic studies at baseline (B) PCA of proteomic studies after insulin treatment (10 μg/mL medium over 3 h) (C) Volcano plot of proteomic studies of Oma1del podocytes at baseline (D) Volcano plot of proteomic studies of Phb2kd podocytes at baseline (E) Volcano plot of proteomic studies of Oma1del podocytes after insulin treatment (10 μg/mL medium over 3 h) (F) Volcano plot of proteomic studies of Phb2kd podocytes after insulin treatment (10 μg/mL medium over 3 h).

**Figure S4:** linked to Figures 1, 2, 3, 4, 5 (A) PCA of metabolome studies (amines) at baseline (B) PCA of metabolome studies (amines) after insulin treatment (10 μg/mL medium over 3 h) (C) PCA of metabolome studies (anionic) at baseline (D) PCA of metabolome studies (anionic) after insulin treatment (10 μg/mL medium over 3 h) (E) PCA of stable isotope enrichment studies of Oma1del podocytes before and after treatment of insulin (10 μg/mL medium over 3 h) with U‐13C6 glucose for 120 min (amines). (F) PCA of stable isotope enrichment studies of Phb2kd podocytes before and after treatment of insulin (10 μg/mL medium over 3 h) with U‐13C6 glucose for 120 min (amines). (G) PCA of stable isotope enrichment studies of Phb2kd podocytes before and after treatment of insulin (10 μg/mL medium over 3 h) with U‐13C5 glutamine for 120 min (amines). (H) PCA of stable isotope enrichment studies of Oma1del podocytes before and after treatment of insulin (10 μg/mL medium over 3 h) with U‐13C6 glucose for 120 min (anionic). (I) PCA of stable isotope enrichment studies of Phb2kd podocytes before and after treatment of insulin (10 μg/mL medium over 3 h) with U‐13C6 glucose for 120 min (anionic). (J) PCA of stable isotope enrichment studies of Phb2kd podocytes before and after treatment of insulin (10 μg/mL medium over 3 h) with U‐13C5 glutamine for 120 min (anionic).

**Figure S5:** linked to Figure 1 (A–E) Stable isotope enrichment studies after treatment of cells with U‐13C6 glucose for 120 min. Data are presented as log2FC versus control ± SEM. Statistical significance was determined using Student's *t*‐test. *p < 0.05; **p < 0.01; ***p < 0.001; ****p < 0.0001. *n* = 4 for all experimental groups. G6P, glucose‐6‐phosphate; F6P, fructose‐6‐phosphate; FBP, fructose‐1,6‐bisphosphate; PA, pyruvic acid; LA, lactic acid.

## Data Availability

All data supporting the findings of this study are available within the article or its Table S1 and Figures [Link], [Link], [Link], [Link], [Link]. Proteomics data of mouse podocytes have been deposited in the BioStudies database (http://www.ebi.ac.uk/biostudies) under accession S‐BSST1760. Metabolomics data are available at BioStudies accession S‐BSST1757 (baseline) and S‐BSST1758 (insulin‐treated). Stable isotope tracing data have been deposited under accession S‐BSST1759.

## References

[1] H. Pavenstadt , W. Kriz , and M. Kretzler , “Cell Biology of the Glomerular Podocyte,” Physiological Reviews 83, no. 1 (2003): 253–307. 12506131 10.1152/physrev.00020.2002

[2] C. J. May , M. Saleem , and G. I. Welsh , “Podocyte Dedifferentiation: A Specialized Process for a Specialized Cell,” Frontiers in Endocrinology 5 (2014): 148. 25324828 10.3389/fendo.2014.00148 PMC4181233

[3] A. Z. Rosenberg and J. B. Kopp , “Focal Segmental Glomerulosclerosis,” Clinical Journal of the American Society of Nephrology: CJASN 12, no. 3 (2017): 502–517. 28242845 10.2215/CJN.05960616 PMC5338705

[4] J. Reiser and M. M. Altintas , “Podocytes,” F1000Research 5 (2016): 5. 10.12688/f1000research.7255.1 PMC4755401 26918173

[5] P. T. Brinkkoetter , C. Ising , and T. Benzing , “The Role of the Podocyte in Albumin Filtration,” Nature Reviews Nephrology 9, no. 6 (2013): 328–336. 23609563 10.1038/nrneph.2013.78

[6] R. Coward and A. Fornoni , “Insulin Signaling: Implications for Podocyte Biology in Diabetic Kidney Disease,” Current Opinion in Nephrology and Hypertension 24, no. 1 (2015): 104–110. 25415617 10.1097/MNH.0000000000000078 PMC4386894

[7] G. I. Welsh , L. J. Hale , V. Eremina , et al., “Insulin Signaling to the Glomerular Podocyte Is Critical for Normal Kidney Function,” Cell Metabolism 12, no. 4 (2010): 329–340. 20889126 10.1016/j.cmet.2010.08.015 PMC4949331

[8] A. C. Lay and R. J. M. Coward , “The Evolving Importance of Insulin Signaling in Podocyte Health and Disease,” Frontiers in Endocrinology 9 (2018): 693. 30524379 10.3389/fendo.2018.00693 PMC6258712

[9] A. Kuczkowski and P. T. Brinkkoetter , “Metabolism and Homeostasis in the Kidney: Metabolic Regulation Through Insulin Signaling in the Kidney,” Cell and Tissue Research 369, no. 1 (2017): 199–210. 28413863 10.1007/s00441-017-2619-7

[10] R. G. Bonegio , R. Fuhro , Z. Wang , et al., “Rapamycin Ameliorates Proteinuria‐Associated Tubulointerstitial Inflammation and Fibrosis in Experimental Membranous Nephropathy,” Journal of the American Society of Nephrology: JASN 16, no. 7 (2005): 2063–2072. 15917339 10.1681/ASN.2004030180

[11] K. Inoki and T. B. Huber , “Mammalian Target of Rapamycin Signaling in the Podocyte,” Current Opinion in Nephrology and Hypertension 21, no. 3 (2012): 251–257. 22388550 10.1097/MNH.0b013e3283520f38

[12] N. Ito , Y. Nishibori , Y. Ito , et al., “mTORC1 Activation Triggers the Unfolded Protein Response in Podocytes and Leads to Nephrotic Syndrome,” Laboratory Investigation; a Journal of Technical Methods and Pathology 91, no. 11 (2011): 1584–1595. 21876538 10.1038/labinvest.2011.135

[13] R. Naumovic , D. Jovovic , G. Basta‐Jovanovic , et al., “Effects of Rapamycin on Active Heymann Nephritis,” American Journal of Nephrology 27, no. 4 (2007): 379–389. 17570905 10.1159/000103918

[14] G. K. Rangan and J. D. Coombes , “Renoprotective Effects of Sirolimus in Non‐Immune Initiated Focal Segmental Glomerulosclerosis,” Nephrology, Dialysis, Transplantation: Official Publication of the European Dialysis and Transplant Association—European Renal Association 22, no. 8 (2007): 2175–2182. 17550925 10.1093/ndt/gfm191

[15] M. Godel , B. Hartleben , N. Herbach , et al., “Role of mTOR in Podocyte Function and Diabetic Nephropathy in Humans and Mice,” Journal of Clinical Investigation 121, no. 6 (2011): 2197–2209. 21606591 10.1172/JCI44774 PMC3104746

[16] A. Fukuda , M. A. Chowdhury , M. P. Venkatareddy , et al., “Growth‐Dependent Podocyte Failure Causes Glomerulosclerosis,” Journal of the American Society of Nephrology: JASN 23, no. 8 (2012): 1351–1363. 22773827 10.1681/ASN.2012030271 PMC3402293

[17] K. Inoki , H. Mori , J. Wang , et al., “mTORC1 Activation in Podocytes Is a Critical Step in the Development of Diabetic Nephropathy in Mice,” Journal of Clinical Investigation 121, no. 6 (2011): 2181–2196. 21606597 10.1172/JCI44771 PMC3104745

[18] A. C. Lay , J. A. Hurcombe , V. M. S. Betin , et al., “Prolonged Exposure of Mouse and Human Podocytes to Insulin Induces Insulin Resistance Through Lysosomal and Proteasomal Degradation of the Insulin Receptor,” Diabetologia 60, no. 11 (2017): 2299–2311. 28852804 10.1007/s00125-017-4394-0 PMC6448913

[19] R. J. Coward , G. I. Welsh , A. Koziell , et al., “Nephrin Is Critical for the Action of Insulin on Human Glomerular Podocytes,” Diabetes 56, no. 4 (2007): 1127–1135. 17395751 10.2337/db06-0693

[20] S. Liu , Y. Yuan , Y. Xue , C. Xing , and B. Zhang , “Podocyte Injury in Diabetic Kidney Disease: A Focus on Mitochondrial Dysfunction,” Frontiers in Cell and Development Biology 10 (2022): 832887. 10.3389/fcell.2022.832887 PMC8935076 35321238

[21] L. Tilokani , S. Nagashima , V. Paupe , and J. Prudent , “Mitochondrial Dynamics: Overview of Molecular Mechanisms,” Essays in Biochemistry 62, no. 3 (2018): 341–360. 30030364 10.1042/EBC20170104 PMC6056715

[22] C. Merkwirth , S. Dargazanli , T. Tatsuta , et al., “Prohibitins Control Cell Proliferation and Apoptosis by Regulating OPA1‐Dependent Cristae Morphogenesis in Mitochondria,” Genes & Development 22, no. 4 (2008): 476–488. 18281461 10.1101/gad.460708 PMC2238669

[23] C. Osman , C. Merkwirth , and T. Langer , “Prohibitins and the Functional Compartmentalization of Mitochondrial Membranes,” Journal of Cell Science 122, no. Pt 21 (2009): 3823–3830. 19889967 10.1242/jcs.037655

[24] M. J. Baker , P. A. Lampe , D. Stojanovski , et al., “Stress‐Induced OMA1 Activation and Autocatalytic Turnover Regulate OPA1‐Dependent Mitochondrial Dynamics,” EMBO Journal 33, no. 6 (2014): 578–593. 24550258 10.1002/embj.201386474 PMC3989652

[25] S. Ehses , I. Raschke , G. Mancuso , et al., “Regulation of OPA1 Processing and Mitochondrial Fusion by m‐AAA Protease Isoenzymes and OMA1,” Journal of Cell Biology 187, no. 7 (2009): 1023–1036. 20038678 10.1083/jcb.200906084 PMC2806285

[26] B. Head , L. Griparic , M. Amiri , S. Gandre‐Babbe , and A. M. van der Bliek , “Inducible Proteolytic Inactivation of OPA1 Mediated by the OMA1 Protease in Mammalian Cells,” Journal of Cell Biology 187, no. 7 (2009): 959–966. 20038677 10.1083/jcb.200906083 PMC2806274

[27] N. Ishihara , Y. Fujita , T. Oka , and K. Mihara , “Regulation of Mitochondrial Morphology Through Proteolytic Cleavage of OPA1,” EMBO Journal 25, no. 13 (2006): 2966–2977. 16778770 10.1038/sj.emboj.7601184 PMC1500981

[28] S. Ozawa , S. Ueda , H. Imamura , et al., “Glycolysis, but Not Mitochondria, Responsible for Intracellular ATP Distribution in Cortical Area of Podocytes,” Scientific Reports 5 (2015): 18575. 26677804 10.1038/srep18575 PMC4683464

[29] J. M. Forbes and D. R. Thorburn , “Mitochondrial Dysfunction in Diabetic Kidney Disease,” Nature Reviews Nephrology 14, no. 5 (2018): 291–312. 29456246 10.1038/nrneph.2018.9

[30] X. Fan , M. Yang , Y. Lang , et al., “Mitochondrial Metabolic Reprogramming in Diabetic Kidney Disease,” Cell Death & Disease 15, no. 6 (2024): 442. 38910210 10.1038/s41419-024-06833-0 PMC11194272

[31] C. Ising , S. Koehler , S. Brahler , et al., “Inhibition of Insulin/IGF‐1 Receptor Signaling Protects From Mitochondria‐Mediated Kidney Failure,” EMBO Molecular Medicine 7, no. 3 (2015): 275–287. 25643582 10.15252/emmm.201404916 PMC4364945

[32] M. A. Saleem , M. J. O'Hare , J. Reiser , et al., “A Conditionally Immortalized Human Podocyte Cell Line Demonstrating Nephrin and Podocin Expression,” Journal of the American Society of Nephrology: JASN 13, no. 3 (2002): 630–638. 11856766 10.1681/ASN.V133630

[33] J. Lam , P. Katti , M. Biete , et al., “A Universal Approach to Analyzing Transmission Electron Microscopy With ImageJ,” Cells 10, no. 9 (2021): 2177. 34571826 10.3390/cells10092177 PMC8465115

[34] M. M. Rinschen , X. Wu , T. Konig , et al., “Phosphoproteomic Analysis Reveals Regulatory Mechanisms at the Kidney Filtration Barrier,” Journal of the American Society of Nephrology 25, no. 7 (2014): 1509–1522. 24511133 10.1681/ASN.2013070760 PMC4073431

[35] M. M. Rinschen , M. J. Yu , G. Wang , et al., “Quantitative Phosphoproteomic Analysis Reveals Vasopressin V2‐Receptor‐Dependent Signaling Pathways in Renal Collecting Duct Cells,” Proceedings of the National Academy of Sciences of the United States of America 107, no. 8 (2010): 3882–3887. 20139300 10.1073/pnas.0910646107 PMC2840509

[36] C. D. Kelstrup , C. Young , R. Lavallee , M. L. Nielsen , and J. V. Olsen , “Optimized Fast and Sensitive Acquisition Methods for Shotgun Proteomics on a Quadrupole Orbitrap Mass Spectrometer,” Journal of Proteome Research 11, no. 6 (2012): 3487–3497. 22537090 10.1021/pr3000249

[37] X. Su , W. Lu , and J. D. Rabinowitz , “Metabolite Spectral Accuracy on Orbitraps,” Analytical Chemistry 89, no. 11 (2017): 5940–5948. 28471646 10.1021/acs.analchem.7b00396 PMC5748891

[38] Z. Wu , C. Xiao , F. Li , W. Huang , F. You , and X. Li , “Mitochondrial Fusion‐Fission Dynamics and Its Involvement in Colorectal Cancer,” Molecular Oncology 18, no. 5 (2024): 1058–1075. 38158734 10.1002/1878-0261.13578 PMC11076987

[39] T. Tatsuta and T. Langer , “Prohibitins,” Current Biology: CB 27, no. 13 (2017): R629–R631. 28697355 10.1016/j.cub.2017.04.030

[40] S. Cogliati , J. A. Enriquez , and L. Scorrano , “Mitochondrial Cristae: Where Beauty Meets Functionality,” Trends in Biochemical Sciences 41, no. 3 (2016): 261–273. 26857402 10.1016/j.tibs.2016.01.001

[41] C. Yang , B. Ko , C. T. Hensley , et al., “Glutamine Oxidation Maintains the TCA Cycle and Cell Survival During Impaired Mitochondrial Pyruvate Transport,” Molecular Cell 56, no. 3 (2014): 414–424. 25458842 10.1016/j.molcel.2014.09.025 PMC4268166

[42] P. Forny , X. Bonilla , D. Lamparter , et al., “Integrated Multi‐Omics Reveals Anaplerotic Rewiring in Methylmalonyl‐CoA Mutase Deficiency,” Nature Metabolism 5, no. 1 (2023): 80–95. 10.1038/s42255-022-00720-8 PMC9886552 36717752

[43] S. J. Shankland , J. W. Pippin , J. Reiser , and P. Mundel , “Podocytes in Culture: Past, Present, and Future,” Kidney International 72, no. 1 (2007): 26–36. 17457377 10.1038/sj.ki.5002291

[44] M. D. Brand and D. G. Nicholls , “Assessing Mitochondrial Dysfunction in Cells,” Biochemical Journal 435, no. 2 (2011): 297–312. 21726199 10.1042/BJ20110162 PMC3076726

[45] A. R. Mullen , W. W. Wheaton , E. S. Jin , et al., “Reductive Carboxylation Supports Growth in Tumour Cells With Defective Mitochondria,” Nature 481, no. 7381 (2011): 385–388. 22101431 10.1038/nature10642 PMC3262117

[46] C. E. Geisler , S. Ghimire , S. M. Bruggink , et al., “A Critical Role of Hepatic GABA in the Metabolic Dysfunction and Hyperphagia of Obesity,” Cell Reports 35, no. 13 (2021): 109301. 34192532 10.1016/j.celrep.2021.109301 PMC8851954

[47] Z. Wu , M. Zuo , L. Zeng , et al., “OMA1 Reprograms Metabolism Under Hypoxia to Promote Colorectal Cancer Development,” EMBO Reports 22, no. 1 (2021): e50827. 33314701 10.15252/embr.202050827 PMC7788456

[48] P. Rivera‐Mejias , A. J. Narbona‐Perez , L. Hasberg , et al., “The Mitochondrial Protease OMA1 Acts as a Metabolic Safeguard Upon Nuclear DNA Damage,” Cell Reports 42, no. 4 (2023): 112332. 37002921 10.1016/j.celrep.2023.112332

[49] J. Fu , T. Shinjo , Q. Li , et al., “Regeneration of Glomerular Metabolism and Function by Podocyte Pyruvate Kinase M2 in Diabetic Nephropathy,” JCI Insight 7, no. 5 (2022): e155260. 35133981 10.1172/jci.insight.155260 PMC8983139

[50] A. Korwitz , C. Merkwirth , R. Richter‐Dennerlein , et al., “Loss of OMA1 Delays Neurodegeneration by Preventing Stress‐Induced OPA1 Processing in Mitochondria,” Journal of Cell Biology 212, no. 2 (2016): 157–166. 26783299 10.1083/jcb.201507022 PMC4738383

[51] X. Xiao , Y. Hu , P. M. Quiros , Q. Wei , C. Lopez‐Otin , and Z. Dong , “OMA1 Mediates OPA1 Proteolysis and Mitochondrial Fragmentation in Experimental Models of Ischemic Kidney Injury,” American Journal of Physiology. Renal Physiology 306, no. 11 (2014): F1318–F1326. 24671334 10.1152/ajprenal.00036.2014 PMC4042105

[52] Q. Yang , W. Xie , X. Wang , et al., “SS31 Ameliorates Podocyte Injury via Inhibiting OMA1‐Mediated Hydrolysis of OPA1 in Diabetic Kidney Disease,” Frontiers in Pharmacology 12 (2021): 707006. 36338294 10.3389/fphar.2021.707006 PMC9629008

[53] P. M. Quiros , A. J. Ramsay , D. Sala , et al., “Loss of Mitochondrial Protease OMA1 Alters Processing of the GTPase OPA1 and Causes Obesity and Defective Thermogenesis in Mice,” EMBO Journal 31, no. 9 (2012): 2117–2133. 22433842 10.1038/emboj.2012.70 PMC3343468

[54] S. Ahola , P. Rivera Mejias , S. Hermans , et al., “OMA1‐Mediated Integrated Stress Response Protects Against Ferroptosis in Mitochondrial Cardiomyopathy,” Cell Metabolism 34, no. 11 (2022): 1875–1891. 36113464 10.1016/j.cmet.2022.08.017

