# Supplementary material for: Disruption of Mitochondrial Dynamics and Integrity Drives Divergent Metabolic Flexibility and Resilience in Podocytes
Source: FASEB J. 2025 Dec 13;39(24):e71340. doi: 10.1096/fj.202502934R (PMC12701524; doi:10.1096/fj.202502934R)
Supplement: Supplementary file 1 — Table S1: linked to Figures 1, 2, 3, 4, 5: log2FC and FDR of proteomic studies of enzymes of glycolysis before and after insulin treatment (10 μg/mL medium over 3 h). ALDOA, aldolase a; ENO1, enolase; GAPDH, glyceraldehyde‐3‐phosphate dehydrogenase; GPI, glucose‐6‐phosphate isomerase; HK1/2, hexokinase 1/2; LDHA/B, lactate dehydrogenase a/b; PCK2, phosphoenolpyruvate carboxykinase 2 (mitochondrial); PFKLM/P, phosphofructokinase (liver/muscle/platelet); PGAM1, phosphoglycerate mutase 1; PGK1, phosphoglycerate kinase; PKM, pyruvate kinase M1/2; TPI1, triosephosphate isomerase 1. ACO2, aconitase 2; CS, citrate synthase; DLD, dihydrolipoamide dehydrogenase; DLST, dihydrolipoamide s‐succinyltransferase; FH, fumarate hydratase; IDH1, cytoplasmic isocitrate dehydrogenase [NADP]; IDH2, mitochondrial isocitrate dehydrogenase [NADP]; IDH3A, isocitrate dehydrogenase (NAD(+)) 3 catalytic subunit alpha; IDH3G, isocitrate dehydrogenase (NAD(+)) 3 non‐catalytic subunit gamma; MDH2, malate dehydrogenase 2; OGDH, oxoglutarate dehydrogenase; SDHA, succinate dehydrogenase complex flavoprotein subunit a; SDHB, succinate dehydrogenase complex iron sulfur subunit b; SUCLA2, succinate‐CoA ligase ADP‐forming subunit beta; SUCLG1, succinate‐CoA ligase GDP/ADP‐forming subunit alpha; SUCLG2, succinate‐CoA ligase GDP/ADP‐forming subunit beta; GLS, glutaminase; GLUL, glutamine synthetase; GLUD1, glutamate dehydrogenase 1; AKT1, RAC‐alpha serine/threonine‐protein kinase; BAD, Bcl2‐associated agonist of cell death; GSK3B, glycogen synthase kinase‐3 beta; IRS2, insulin receptor substrate 2; KRAS, GTPase KRas; MAP2K1/2, dual specificity mitogen‐activated protein kinase kinase 1/2; MAPK3, mitogen‐activated protein kinase 3; PCK2, phosphoenolpyruvate carboxykinase 2 (mitochondrial); PIK3C3, phosphatidylinositol 3‐kinase catalytic subunit type 3; PIK3R1, phosphatidylinositol 3‐kinase regulatory subunit alpha; RAF1, RAF proto‐oncogene serine/threonine‐protein kinase; RPS6, 40S ribosomal protein S6. [file FSB2-39-e71340-s002.docx]

**Supplementary Table 1: linked to Figures 1 – 5**

|  | **baseline** | | | | **insulin-treatment** | | | |
| --- | --- | --- | --- | --- | --- | --- | --- | --- |
|  | **Oma1del** | | **Phb2kd** | | **Oma1del** | | **Phb2kd** | |
| **Protein** | **log2 FC** | **FDR** | **log2 FC** | **FDR** | **log2 FC** | **FDR** | **log2 FC** | **FDR** |
| ALDOA | **0,62** | 0,01 | **-0,48** | 0,16 | **0,34** | 0,17 | **-0,05** | 0,94 |
| ENO1 | **0,29** | 0,12 | **0,27** | 0,30 | **0,40** | 0,08 | **0,21** | 0,69 |
| GAPDH | **0,92** | 0,00 | **0,21** | 0,50 | **1,25** | 0,00 | **0,05** | 0,93 |
| GPI | **0,21** | 0,18 | **0,07** | 0,89 | **0,45** | 0,05 | **0,19** | 0,70 |
| HK1 | **-0,19** | 0,30 | **0,33** | 0,23 | **0,01** | 0,97 | **0,58** | 0,23 |
| HK2 | **1,08** | 0,02 | **0,46** | 0,38 | **1,17** | 0,07 | **1,30** | 0,12 |
| LDHA | **0,38** | 0,05 | **0,09** | 0,84 | **0,85** | 0,01 | **0,40** | 0,50 |
| LDHB | **0,47** | 0,07 | **0,27** | 0,61 | **0,80** | 0,01 | **0,16** | 0,81 |
| PCK2 | **0,47** | 0,07 | **0,27** | 0,61 | **0,80** | 0,01 | **0,16** | 0,81 |
| PFKL | **1,14** | 0,00 | **0,32** | 0,29 | **1,31** | 0,00 | **0,40** | 0,51 |
| PFKM | **0,70** | 0,01 | **-0,23** | 0,66 | **0,58** | 0,05 | **0,40** | 0,27 |
| PFKP | **1,21** | 0,00 | **-1,46** | 0,01 | **1,52** | 0,00 | **-0,32** | 0,65 |
| PGAM1 | **0,47** | 0,04 | **0,33** | 0,37 | **0,67** | 0,02 | **0,45** | 0,27 |
| PGK1 | **0,34** | 0,08 | **-0,02** | 0,96 | **0,64** | 0,01 | **0,18** | 0,74 |
| PKM | **0,12** | 0,53 | **0,04** | 0,93 | **0,26** | 0,26 | **0,09** | 0,87 |
| TPI1 | **0,01** | 0,95 | **-0,09** | 0,85 | **0,16** | 0,50 | **0,17** | 0,67 |
| ACO2 | **-0,51** | 0,01 | **-0,04** | 0,93 | **-0,47** | 0,04 | **-0,04** | 0,95 |
| CS | **0,79** | 0,00 | **-0,44** | 0,30 | **0,98** | 0,01 | **-0,07** | 0,93 |
| DLD | **0,01** | 0,96 | **0,00** | 1,00 | **-0,05** | 0,88 | **-0,01** | 0,99 |
| DLST | **0,28** | 0,22 | **0,28** | 0,65 | **0,05** | 0,89 | **-0,06** | 0,95 |
| FH | **0,07** | 0,71 | **-0,18** | 0,69 | **-0,07** | 0,81 | **-0,34** | 0,61 |
| IDH1 | **0,44** | 0,05 | **0,16** | 0,64 | **0,84** | 0,01 | **0,25** | 0,62 |
| IDH2 | **0,17** | 0,54 | **-0,67** | 0,10 | **-0,20** | 0,46 | **-0,44** | 0,35 |
| IDH3A | **-0,22** | 0,19 | **-0,82** | 0,08 | **-0,28** | 0,21 | **-0,31** | 0,70 |
| IDH3G | **-0,33** | 0,11 | **-0,35** | 0,64 | **-0,17** | 0,56 | **-0,12** | 0,90 |
| MDH2 | **-0,35** | 0,05 | **-0,01** | 1,00 | **-0,48** | 0,05 | **-0,09** | 0,89 |
| OGDH | **-0,45** | 0,02 | **-0,46** | 0,32 | **-0,60** | 0,02 | **-0,20** | 0,77 |
| SDHA | **-0,23** | 0,29 | **-0,41** | 0,25 | **-0,48** | 0,05 | **-0,17** | 0,78 |
| SDHB | **-0,72** | 0,03 | **-0,31** | 0,43 | **-0,21** | 0,38 | **-0,13** | 0,84 |
| SUCLA2 | **0,26** | 0,21 | **-0,23** | 0,71 | **0,36** | 0,14 | **-0,18** | 0,75 |
| SUCLG1 | **0,79** | 0,01 | **-0,87** | 0,09 | **0,29** | 0,38 | **-0,02** | 0,99 |
| SUCLG2 | **0,63** | 0,04 | **-0,08** | 0,87 | **-0,03** | 0,93 | **-0,17** | 0,78 |
| GLS | **1,92** | 0,00 | **0,10** | 0,83 | **1,56** | 0,00 | **-0,17** | 0,82 |
| GLUD1 | **1,52** | 0,00 | **0,10** | 0,87 | **1,32** | 0,01 | **0,21** | 0,62 |
| GLUL | **0,64** | 0,26 | **0,03** | 0,98 | **2,31** | 0,00 | **-0,27** | 0,83 |
| AKT1 | **0,49** | 0,14 | **0,89** | 0,40 | **2,31** | 0,01 | **-0,31** | 0,73 |
| BAD | **0,54** | 0,11 | **-0,31** | 0,52 | **1,07** | 0,03 | **0,04** | 0,96 |
| GSK3B | **-0,67** | 0,02 | **0,17** | 0,74 | **-0,76** | 0,03 | **0,16** | 0,82 |
| IRS2 | **2,27** | 0,00 | **-0,08** | 0,87 | **1,47** | 0,00 | **0,00** | 1,00 |
| KRAS | **1,05** | 0,00 | **-0,55** | 0,22 | **0,99** | 0,03 | **-0,41** | 0,78 |
| MAP2K1 | **0,11** | 0,64 | **0,08** | 0,81 | **-0,29** | 0,23 | **0,45** | 0,44 |
| MAP2K2 | **0,59** | 0,04 | **-0,27** | 0,58 | **0,13** | 0,61 | **0,32** | 0,63 |
| MAPK1 | **-0,39** | 0,09 | **-0,11** | 0,81 | **-0,41** | 0,18 | **0,22** | 0,76 |
| MAPK3 | **-0,12** | 0,53 | **-0,49** | 0,27 | **-0,17** | 0,52 | **0,22** | 0,78 |
| PCK2 | **0,47** | 0,07 | **0,27** | 0,61 | **0,80** | 0,01 | **0,16** | 0,81 |
| PIK3C3 | **0,56** | 0,07 | **-1,13** | 0,10 | **0,00** | 1,00 | **-1,27** | 0,21 |
| PIK3R1 | **-2,03** | 0,00 | **-0,11** | 0,78 | **-0,36** | 0,32 | **0,21** | 0,75 |
| RAF1 | **0,21** | 0,43 | **-0,37** | 0,39 | **0,00** | 1,00 | **0,32** | 0,61 |
| RPS6 | **-0,02** | 0,95 | **-0,01** | 0,98 | **-0,02** | 0,97 | **0,33** | 0,59 |

log2FC and FDR of proteomic studies of enzymes of glycolysis before and after insulin treatment (10 µg/ml medium over 3h). ALDOA: aldolase a, ENO1: enolase, GAPDH: glyceraldehyde-3-phosphate dehydrogenase, GPI: glucose-6-phosphate isomerase, HK1/2: hexokinase 1/2, LDHA/B: lactate dehydrogenase a/b, PCK2: phosphoenolpyruvate carboxykinase 2 (mitochondrial), PFKLM/P: phosphofructokinase (liver/muscle/platelet), PGAM1: phosphoglycerate mutase 1, PGK1: phosphoglycerate kinase, PKM: pyruvate kinase M1/2, TPI1: Triosephosphate Isomerase 1. ACO2: aconitase 2, CS: citrate synthase, DLD: dihydrolipoamide dehydrogenase, DLST: dihydrolipoamide s-succinyltransferase, FH: fumarate hydratase, IDH1: cytoplasmic isocitrate dehydrogenase [NADP], IDH2: mitochondrial isocitrate dehydrogenase [NADP], IDH3A: isocitrate dehydrogenase (NAD(+)) 3 catalytic subunit alpha, IDH3G: isocitrate dehydrogenase (NAD(+)) 3 non-catalytic subunit gamma, MDH2: malate dehydrogenase 2, OGDH: oxoglutarate dehydrogenase, SDHA: succinate dehydrogenase complex flavoprotein subunit a, SDHB: succinate dehydrogenase complex iron sulfur subunit b, SUCLA2: succinate-CoA ligase ADP-forming subunit beta, SUCLG1: succinate-CoA ligase GDP/ADP-forming subunit alpha, SUCLG2: succinate-CoA ligase GDP/ADP-forming subunit beta. GLS: glutaminase, GLUL: glutamine synthetase, GLUD1: glutamate dehydrogenase 1. AKT1: RAC-alpha serine/threonine-protein kinase, BAD: Bcl2-associated agonist of cell death, GSK3B: glycogen synthase kinase-3 beta, IRS2: Insulin receptor substrate 2, KRAS: GTPase KRas, MAP2K1/2: dual specificity mitogen-activated protein kinase kinase 1/2, MAPK3: Mitogen-activated protein kinase 3, PCK2: phosphoenolpyruvate carboxykinase 2 (mitochondrial), PIK3C3: phosphatidylinositol 3-kinase catalytic subunit type 3, PIK3R1: phosphatidylinositol 3-kinase regulatory subunit alpha, RAF1: RAF proto-oncogene serine/threonine-protein kinase, RPS6: 40S ribosomal protein S6
