# Supplementary figures and images for: Disruption of Mitochondrial Dynamics and Integrity Drives Divergent Metabolic Flexibility and Resilience in Podocytes
Source: FASEB J. 2025 Dec 13;39(24):e71340. doi: 10.1096/fj.202502934R (PMC12701524; doi:10.1096/fj.202502934R)

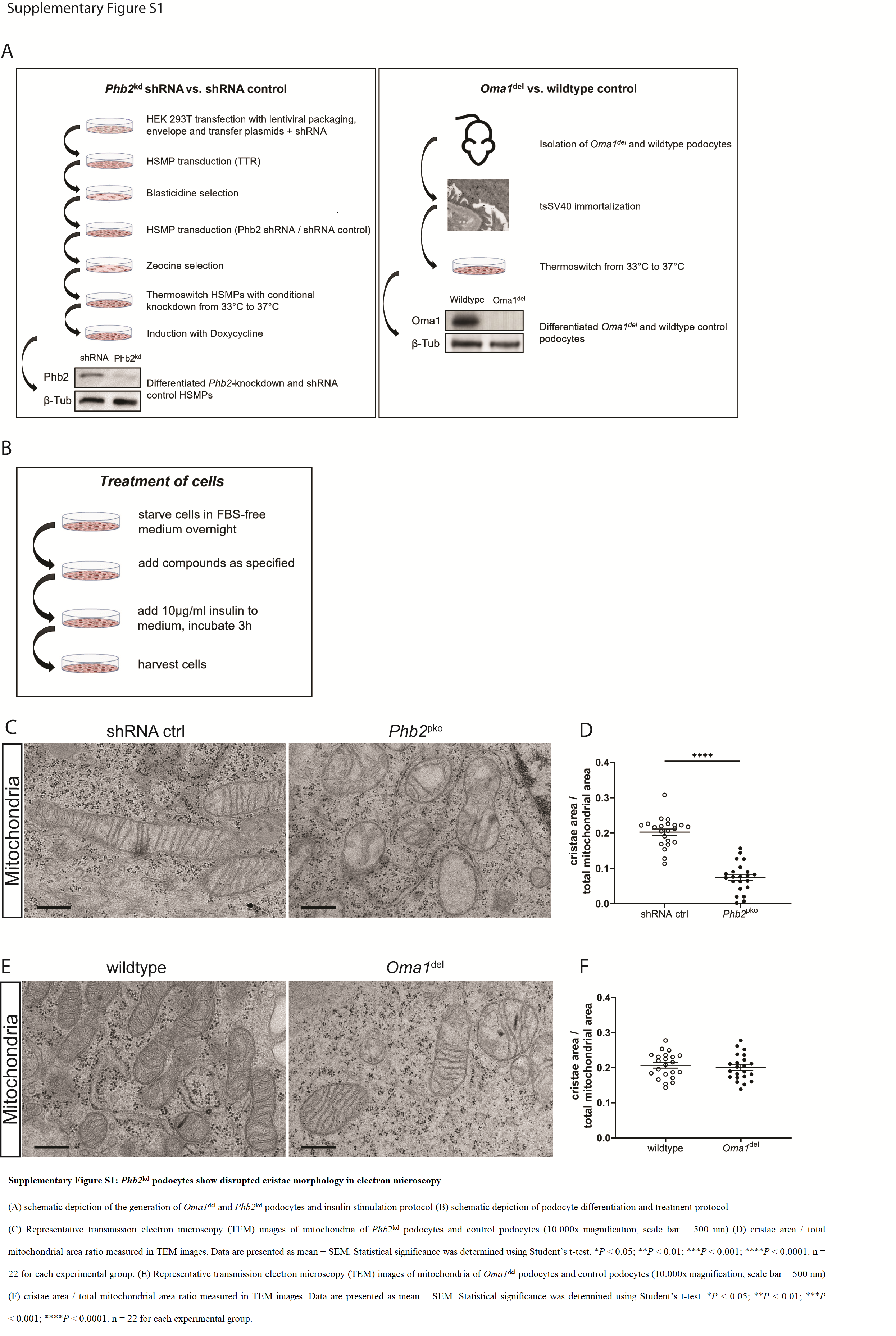

Supplement: Supplementary file 2 — Figure S1: Phb2kd podocytes show disrupted cristae morphology in electron microscopy (A) schematic depiction of the generation of Oma1del and Phb2kd podocytes and insulin stimulation protocol (B) schematic depiction of podocyte differentiation and treatment protocol (C) Representative transmission electron microscopy (TEM) images of mitochondria of Phb2kd podocytes and control podocytes (10.000× magnification, scale bar = 500 nm) (D) cristae area/total mitochondrial area ratio measured in TEM images. Data are presented as mean ± SEM. Statistical significance was determined using Student's t‐test. *p < 0.05; **p < 0.01; ***p < 0.001; ****p < 0.0001. n = 22 for each experimental group. (E) Representative transmission electron microscopy (TEM) images of mitochondria of Oma1del podocytes and control podocytes (10.000× magnification, scale bar = 500 nm) (F) cristae area/total mitochondrial area ratio measured in TEM images. Data are presented as mean ± SEM. Statistical significance was determined using Student's t‐test. *p < 0.05; **p < 0.01; ***p < 0.001; ****p < 0.0001. n = 22 for each experimental group. [file FSB2-39-e71340-s001.tif]

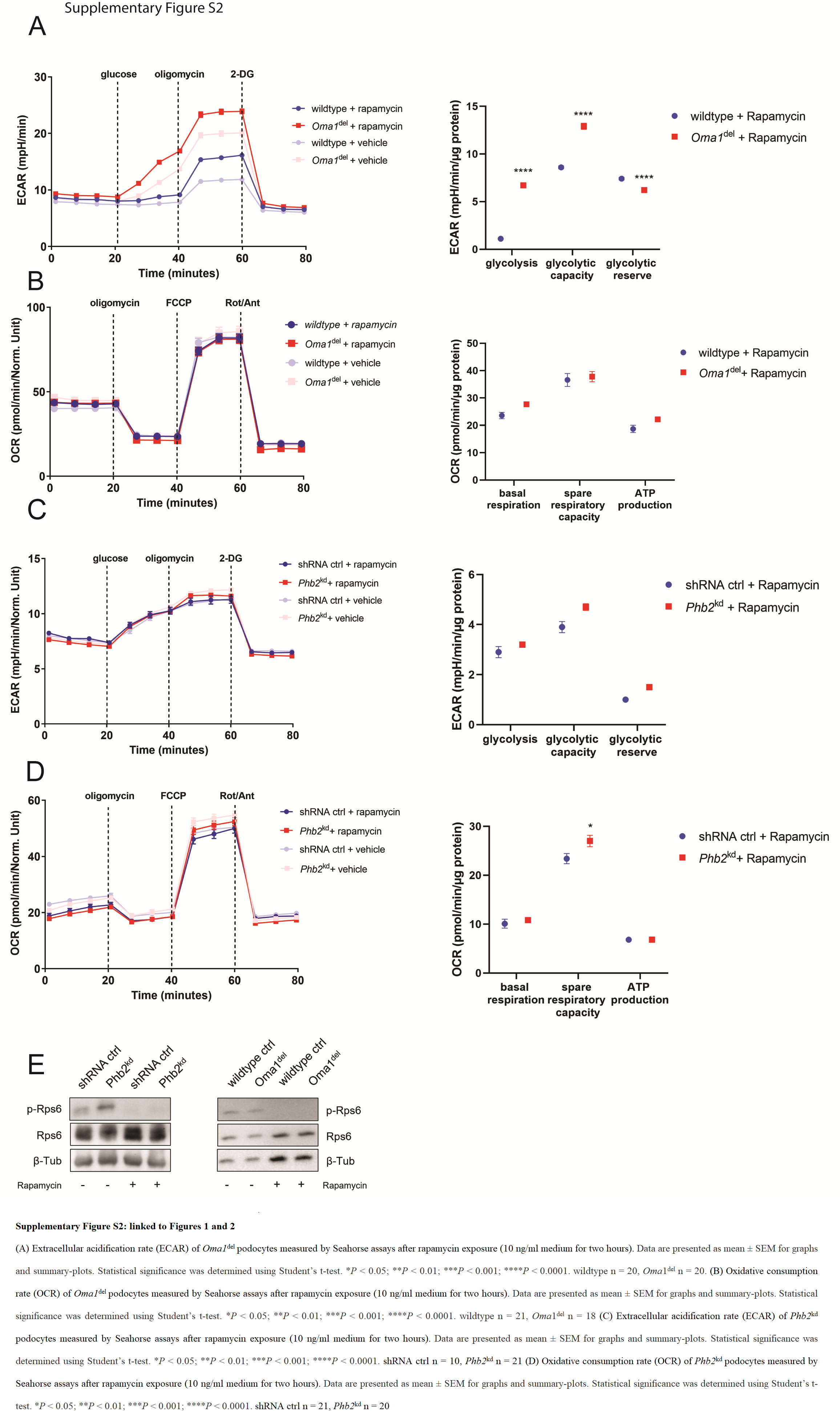

Supplement: Supplementary file 3 — Figure S2: linked to Figures 1 and 2 (A) Extracellular acidification rate (ECAR) of Oma1del podocytes measured by Seahorse assays after rapamycin exposure (10 ng/mL medium for 2 h). Data are presented as mean ± SEM for graphs and summary‐plots. Statistical significance was determined using Student's t‐test. *p < 0.05; **p < 0.01; ***p < 0.001; ****p < 0.0001. Wildtype n = 20, Oma1del n = 20. (B) Oxidative consumption rate (OCR) of Oma1del podocytes measured by Seahorse assays after rapamycin exposure (10 ng/mL medium for 2 h). Data are presented as mean ± SEM for graphs and summary‐plots. Statistical significance was determined using Student's t‐test. *p < 0.05; **p < 0.01; ***p < 0.001; ****p < 0.0001. Wildtype n = 21, Oma1del n = 18. (C) Extracellular acidification rate (ECAR) of Phb2kd podocytes measured by Seahorse assays after rapamycin exposure (10 ng/mL medium for 2 h). Data are presented as mean ± SEM for graphs and summary‐plots. Statistical significance was determined using Student's t‐test. *p < 0.05; **p < 0.01; ***p < 0.001; ****p < 0.0001. shRNA ctrl n = 10, Phb2kd n = 21. (D) Oxidative consumption rate (OCR) of Phb2kd podocytes measured by Seahorse assays after rapamycin exposure (10 ng/mL medium for 2 h). Data are presented as mean ± SEM for graphs and summary‐plots. Statistical significance was determined using Student's t‐test. *p < 0.05; **p < 0.01; ***p < 0.001; ****p < 0.0001. shRNA ctrl n = 21, Phb2kd n = 20. [file FSB2-39-e71340-s003.tif]

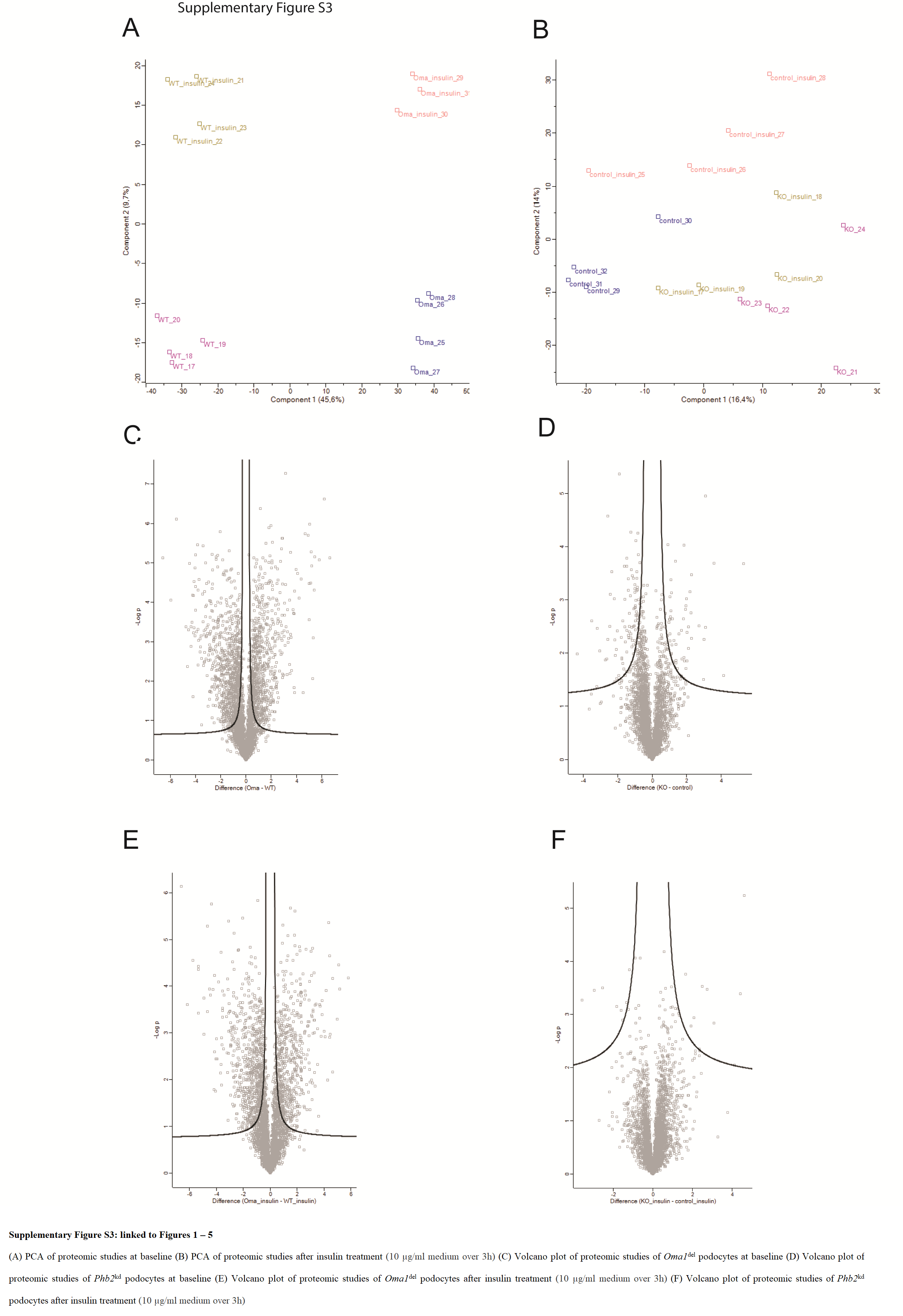

Supplement: Supplementary file 4 — Figure S3: linked to Figures 1, 2, 3, 4, 5 (A) PCA of proteomic studies at baseline (B) PCA of proteomic studies after insulin treatment (10 μg/mL medium over 3 h) (C) Volcano plot of proteomic studies of Oma1del podocytes at baseline (D) Volcano plot of proteomic studies of Phb2kd podocytes at baseline (E) Volcano plot of proteomic studies of Oma1del podocytes after insulin treatment (10 μg/mL medium over 3 h) (F) Volcano plot of proteomic studies of Phb2kd podocytes after insulin treatment (10 μg/mL medium over 3 h). [file FSB2-39-e71340-s006.tif]

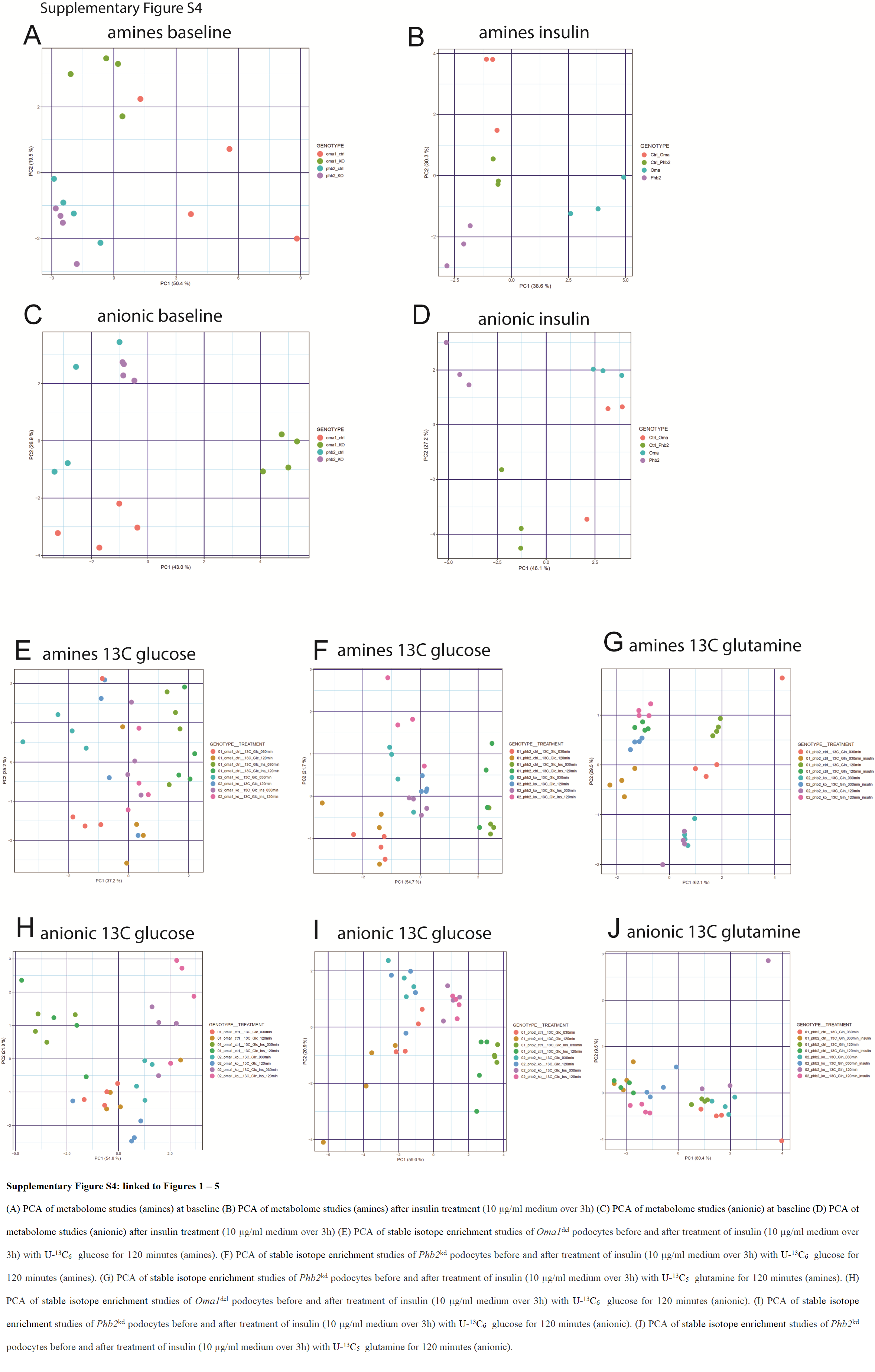

Supplement: Supplementary file 5 — Figure S4: linked to Figures 1, 2, 3, 4, 5 (A) PCA of metabolome studies (amines) at baseline (B) PCA of metabolome studies (amines) after insulin treatment (10 μg/mL medium over 3 h) (C) PCA of metabolome studies (anionic) at baseline (D) PCA of metabolome studies (anionic) after insulin treatment (10 μg/mL medium over 3 h) (E) PCA of stable isotope enrichment studies of Oma1del podocytes before and after treatment of insulin (10 μg/mL medium over 3 h) with U‐13C6 glucose for 120 min (amines). (F) PCA of stable isotope enrichment studies of Phb2kd podocytes before and after treatment of insulin (10 μg/mL medium over 3 h) with U‐13C6 glucose for 120 min (amines). (G) PCA of stable isotope enrichment studies of Phb2kd podocytes before and after treatment of insulin (10 μg/mL medium over 3 h) with U‐13C5 glutamine for 120 min (amines). (H) PCA of stable isotope enrichment studies of Oma1del podocytes before and after treatment of insulin (10 μg/mL medium over 3 h) with U‐13C6 glucose for 120 min (anionic). (I) PCA of stable isotope enrichment studies of Phb2kd podocytes before and after treatment of insulin (10 μg/mL medium over 3 h) with U‐13C6 glucose for 120 min (anionic). (J) PCA of stable isotope enrichment studies of Phb2kd podocytes before and after treatment of insulin (10 μg/mL medium over 3 h) with U‐13C5 glutamine for 120 min (anionic). [file FSB2-39-e71340-s005.tif]

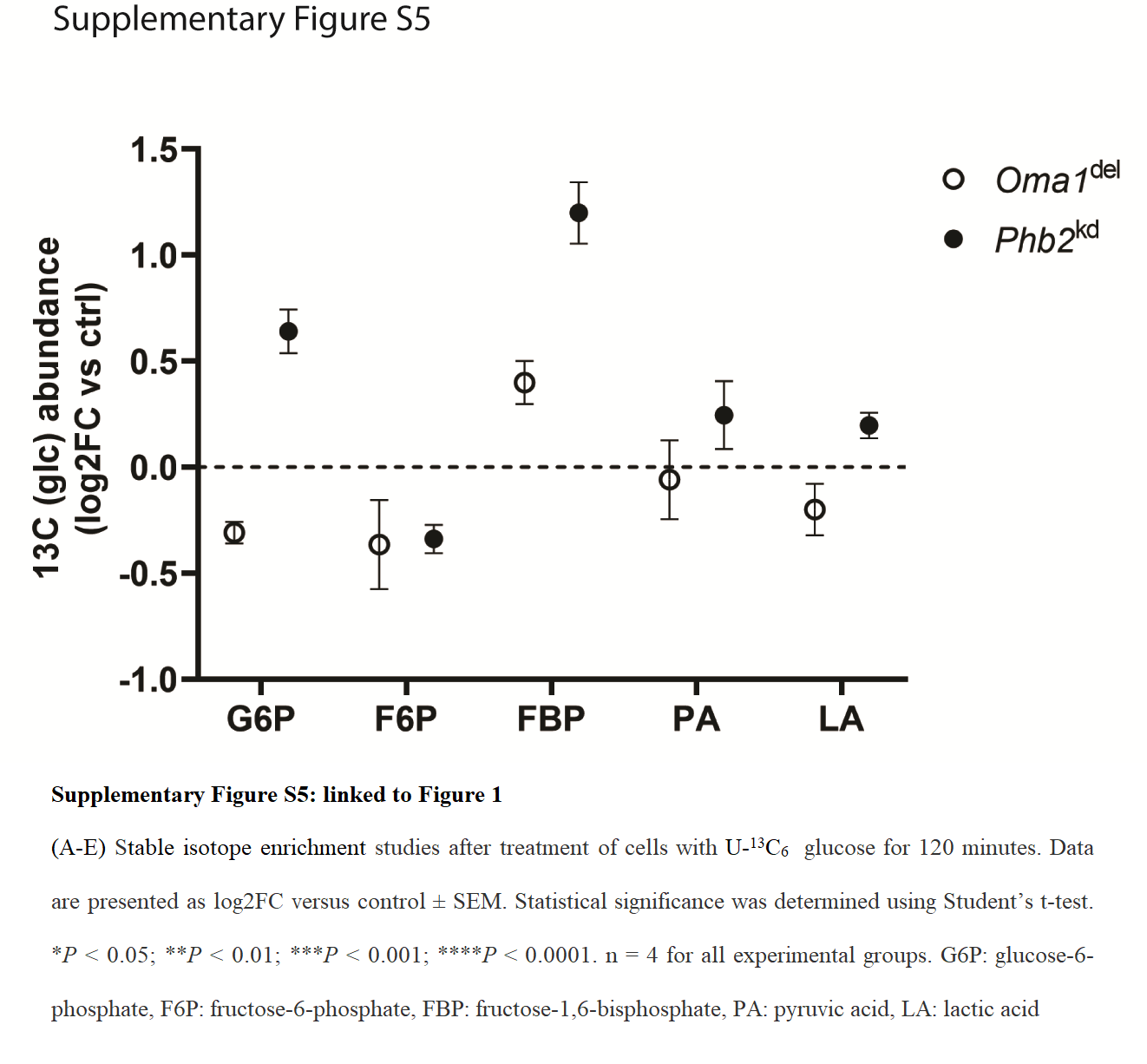

Supplement: Supplementary file 6 — Figure S5: linked to Figure 1 (A–E) Stable isotope enrichment studies after treatment of cells with U‐13C6 glucose for 120 min. Data are presented as log2FC versus control ± SEM. Statistical significance was determined using Student's t‐test. *p < 0.05; **p < 0.01; ***p < 0.001; ****p < 0.0001. n = 4 for all experimental groups. G6P, glucose‐6‐phosphate; F6P, fructose‐6‐phosphate; FBP, fructose‐1,6‐bisphosphate; PA, pyruvic acid; LA, lactic acid. [file FSB2-39-e71340-s004.tif]
